# TAT-CRE inhalation enables tumor induction corresponding to adenoviral Cre-recombinase in a lung cancer mouse model

**DOI:** 10.1038/s42003-025-08146-0

**Published:** 2025-05-13

**Authors:** Tabea Gewalt, Anna M. Dmitrieva, Felix Elsner, Xinlei Zhao, Daniel Dimitri Sieber, Ilayda Gülsen Kocak, Qian Yang, Claudia Viktoria Orschel, Naja Maria Eckert, Bianca Goebel, Marieke Nill, Franziska Peter, Arndt Hartmann, Filippo Beleggia, Margarete Odenthal, Hans Christian Reinhardt, Roland Tillmann Ullrich, Frederik Graw, Lydia Meder

**Affiliations:** 1 Department I of Internal Medicine, Faculty of Medicine and University Hospital Cologne, University of Cologne, Cologne, Germany; 2 Chair of Experimental Medicine I, Medical Faculty, Friedrich-Alexander-Universität Erlangen-Nürnberg (FAU), Erlangen, Germany; 3 Institute of Pathology, Universitätsklinikum Erlangen, Friedrich-Alexander-Universität Erlangen-Nürnberg (FAU), Erlangen, Germany; 4 Institute of Pathology, Faculty of Medicine and University Hospital Cologne, University of Cologne, Cologne, Germany; 5 Department of Translational Genomics, Faculty of Medicine and University Hospital Cologne, University of Cologne, Cologne, Germany; 6 Mildred Scheel School of Oncology Aachen Bonn Cologne Düsseldorf (MSSO ABCD), Faculty of Medicine and University Hospital Cologne, University of Cologne, Cologne, Germany; 7 Department of Hematology and Stem Cell Transplantation, West German Cancer Center, University Hospital Essen, German Cancer Consortium (DKTK), Essen, Germany; 8 Department of Internal Medicine 5, Hematology and Oncology, Friedrich-Alexander-Universität Erlangen-Nürnberg (FAU) and Universitätsklinikum Erlangen, Erlangen, Germany

**Keywords:** Lung cancer, Non-small-cell lung cancer

## Abstract

Cre-recombinase inducible model systems are extensively used in cancer research to manipulate gene expression in specific tissues and induce autochthonous tumor growth. These systems often involve the cross-breeding of genetically engineered organisms containing *loxP*-flanked alleles with those expressing Cre-recombinase. This approach, while effective, has the challenge of requiring high numbers of animals due to breeding requirements. Other frequently used tumor induction methods in cancer research involve the direct application of viral Cre-recombinase vectors. This approach presents the challenge of the accessibility of facilities that meet the necessary safety level. In this context, we perform a comprehensive comparison between TAT-CRE (biosafety level S1) and adenoviral Cre-recombinase induced (biosafety level S2) lung adenocarcinomas driven by *Kras*^*G12D*^ expression and *Trp53* depletion. We use in vivo lung tumor monitoring via computed tomography, single-cell RNA sequencing, immunohistochemistry and flow cytometry to elucidate similarities and differences between TAT-CRE and adenoviral Cre-recombinase induced lung adenocarcinomas. TAT-CRE induced lung tumors present differences in micro-vessels and macrophages but with corresponding tumor onset and growth characteristics compared to adenoviral-Cre recombinase induced lung tumors. Taken together, TAT-CRE is a valuable genetic engineering safety level S1 alternative for cancer induction and may be implemented in other cancer models than lung cancer.

## Abstract (web summary)

A comparison of lung adenocarcinomas induced by TAT-CRE or adenoviral Cre-recombinase revealed similar tumor growth with differences in micro-vessels and macrophages. TAT-CRE offers a valuable S1 alternative for cancer induction in mouse models

## Introduction

Cre-recombinase-inducible model systems are widely used in neurological, immunological, and cancer research^1–3^. Thereby, Cre-recombinase systems are used to express or deplete specific genes of interest in pre-defined tissues or cell types^4^. For this purpose, it is frequently necessary to cross-breed organisms harboring genetically engineered *loxP*-flanked (floxed) exons or stop-codons with organisms delivering the tissue-specific Cre-recombinase activity. As an example, *Amhr2*-Cre strains express Cre-recombinase in the fallopian tubes, uterus and ovaries^5^, *Krt14*-Cre strains enable Cre-recombinase expression in epidermal cells^6^ and the epithelium of the pancreas and duodenum is targeted by using *Pdx1*-Cre strains^7^. This kind of system enables, on the one hand, a specific expression of Cre-recombinase but, on the other hand, requires a high number of animals because of the Cre-stock animal breeding. Regarding the 3Rs principle to reduce, refine and replace animals for experiments, this cross-breeding procedure is demanding and could be improved for some cancer models, such as lung cancer. Moreover, approaches like mini-experiment design and Bayesian updating^8^ can be more easily implemented as a tool to reduce animal numbers.

In cancer research, mouse models are the predominant model system and tumor cell injection models are frequently used^9^. For lung cancer, orthotopic tumor cell injection has been implemented in immuno-deficient^10^ but also in immune-competent mice and has the advantage of rapid tumor induction at the physiological site of tumor origin^11^. However, these models hardly mimic the situation of single pulmonary cells acquiring oncogenic features by accumulation of mutations in protooncogenes or tumor suppressor genes, followed by tumor development from a tumor cell-of-origin, circumventing immunosurveillance and progressing towards a carcinoma^9^. In lung cancer, particularly in lung adenocarcinomas, alveolar type II (ATII) cells expressing surfactant protein C, Club cells expressing CC10 (also known as uteroglobin, encoded by SCGB1A1) and bronchoalveolar stem cells (BASC) positive for CC10 and SPC can serve as tumor cell-of-origin^12–14^.

The acquisition of oncogenic features in cancer models can be addressed by using direct Cre-recombinase application systems. These systems rely frequently on adenoviral- or lentiviral-expressed Cre-recombinase directly applied to the lung^15^, the muscle^16^ or the brain^17^. They have the advantage that the time-point of Cre-recombinase activation can be controlled, for example, starting only in adult mice upon injection of virally expressed Cre-recombinase. However, they have the disadvantage, that viral delivery systems require genetic engineering (GE)-biosafety level S2 facilities, which may limit the ability of using these systems based on location and resources.

Thus, we aimed to establish a Cre-recombinase system for in vivo cancer research, which allows site-specific carcinoma induction under GE-biosafety level S1 conditions without the necessary Cre-strain cross-breeding. Moreover, we wanted to use a genetically engineered mouse model relevant to lung cancer research, but with the potential to study different cancer entities, enabled by site-specific tumor induction. Consequently, the *B6.129-Kras*^*tm4Tyj*^ *Trp53*^*tm1Brn*^*/J* mouse model is a suitable system because lung carcinomas and sarcomas have been successfully induced using virally delivered Cre-recombinase by inhalation and intramuscular injection, respectively^18,19^. In addition, *B6.129-Kras*^*tm4Tyj*^ *Trp53*^*tm1Brn*^*/J* mice have been crossed with a mouse strain expressing tamoxifen-inducible Cre-recombinase under the Pdx1 promotor to induce pancreatic cancer^20^.

We applied a Cre-recombinase protein (TAT-CRE) to the lung via inhalation, to induce the expression of the oncogene Kras^G12D^ and deletion of the tumor suppressor *Trp53*, leading to lung adenocarcinomas^19,21^. Compared to other non-viral Cre delivery systems based on glycoprotein-induced nanovesicles called gesicles^22^ or the type VI protein secretion system (T6SS)^23^, respectively, TAT-CRE has the advantage that it has been recently used to drive floxed allele modification in ex vivo pulmonary tissue^24^ and it can induce recombination globally in all cell-types.

TAT-CRE is a recombinant, cell-permeable fusion Cre-recombinase protein that consists of a trans-activator of transcription (TAT) sequence and a nuclear localization sequence (NLS). TAT-CRE is known to catalyze site-specific recombination between two *loxP* DNA sites, so that the mechanism is identical to the adenovirally expressed Cre-recombinase (AD-CRE) and not changed by fusion to TAT^25^, allowing comparability and common interpretation of the results. However, a systematic comparison of TAT-CRE and AD-CRE induced tumors has not been performed so far. We addressed this need and used in vivo lung tumor monitoring by micro-computed tomography (µCT), single-cell RNA-sequencing (scRNA-seq), immunohistochemistry (IHC) and flow cytometry to uncover differences in characteristics and the tumor environment (TME) between the TAT-CRE and AD-CRE induced lung carcinomas. Finally, we showed the suitability of TAT-CRE in vivo to induce lung adenocarcinomas in comparison to AD-CRE, supporting the 3Rs principles, facilitating mini-experiment design and simultaneously overcoming boundaries for research of limited access to GE-biosafety level S2 resources.

## Results

### Lung cancer induction by TAT-CRE showed a tumor growth pattern similar to AD-CRE induced lung cancer

We systematically compared TAT-CRE and AD-CRE induced lung tumors regarding tumor induction and growth characteristics in order to show the applicability of TAT-CRE in lung cancer mouse models. For this purpose, we used a well-established conditional lung adenocarcinoma mouse model driven by a Cre-inducible *Kras*^*G12D*^ mutant and *Trp53* knock out leading to functional depletion of p53^19,21^.

**Fig. 1:**
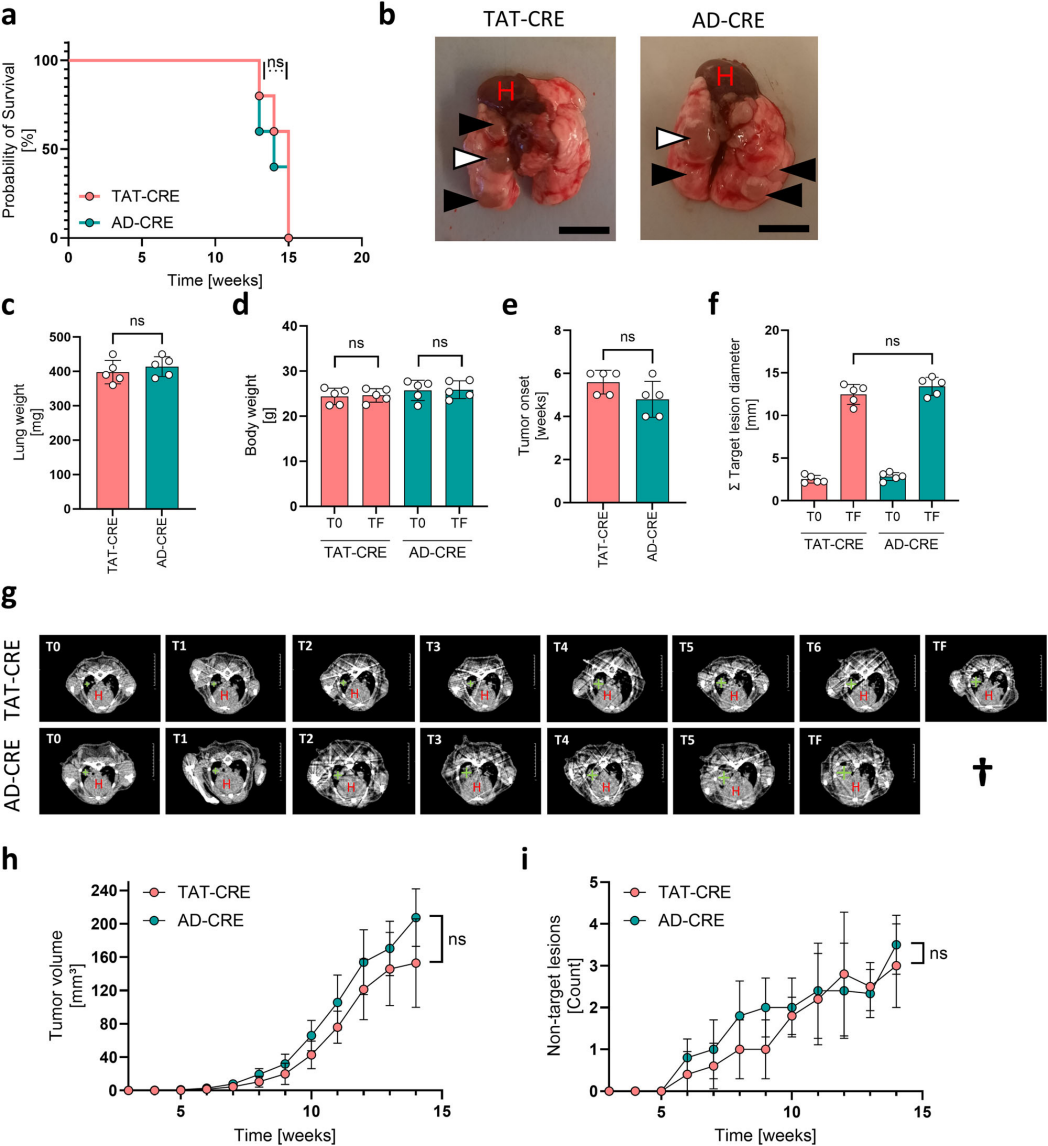
Lung cancer induced by TAT-CRE and AD-CRE showed corresponding macroscopic tumor growth parameters. Lung adenocarcinomas driven by a CRE-inducible conditional Kras^G12D^ mutant and functional Trp53 knock out were induced by TAT-CRE (*n* = 5, 3 males, 2 females) and AD-CRE inhalation (*n* = 5, 3 males, 2 females) and macroscopically analyzed. **a** Probability of survival was analyzed as Kaplan-Meier-Curves and statistically evaluated using the Mantel-Cox test. **b** Picture of final stage lung adenocarcinomas indicating the target lesion (white), the non-target lesions (black) and the heart (H). Bars indicate 8 mm. **c** Lung weight was determined directly after harvest of the lungs. **d** Body weight at T0 (3 weeks after inhalation) and TF (final stage before lung harvest). **e**, **f** Tumor onset and target lesion diameter at T0 (3 weeks after inhalation) and TF (final stage before lung harvest) lesions were determined based on µCT DICOM data analysis using OsiriX. c-f p-values were calculated using an unpaired two-sided t-test. Data is shown as mean ± SD. P-values ≤ 0.05 are considered as significant. **g** Representative weekly µCT measurements starting 3 weeks after CRE inhalation. Target lesion and heart (H) are indicated. **h**, **i** Tumor volume and non-target lesions were determined based on µCT DICOM data analysis using OsiriX. Statistical analysis was done using ANOVA. P-values ≤ 0.05 are considered as significant.

Using TAT-CRE and AD-CRE, we detected similar survival probability and macroscopic lung tumor appearance (Fig. 1a, b). The final lung weight, the total body weight, the tumor onset and the sum of target lesion diameters measured by µCT were comparable between TAT-CRE and AD-CRE induced lung tumors (Fig. 1c–g). µCT is a minimal invasive detection method enabling tumor volume calculation of the primary lesion in vivo and counting the secondary not-target lesions in the lung in vivo over time. For both parameters, we did not detect a significant difference upon TAT-CRE vs AD-CRE tumor induction (Fig. 1h, i).

**Fig. 2:**
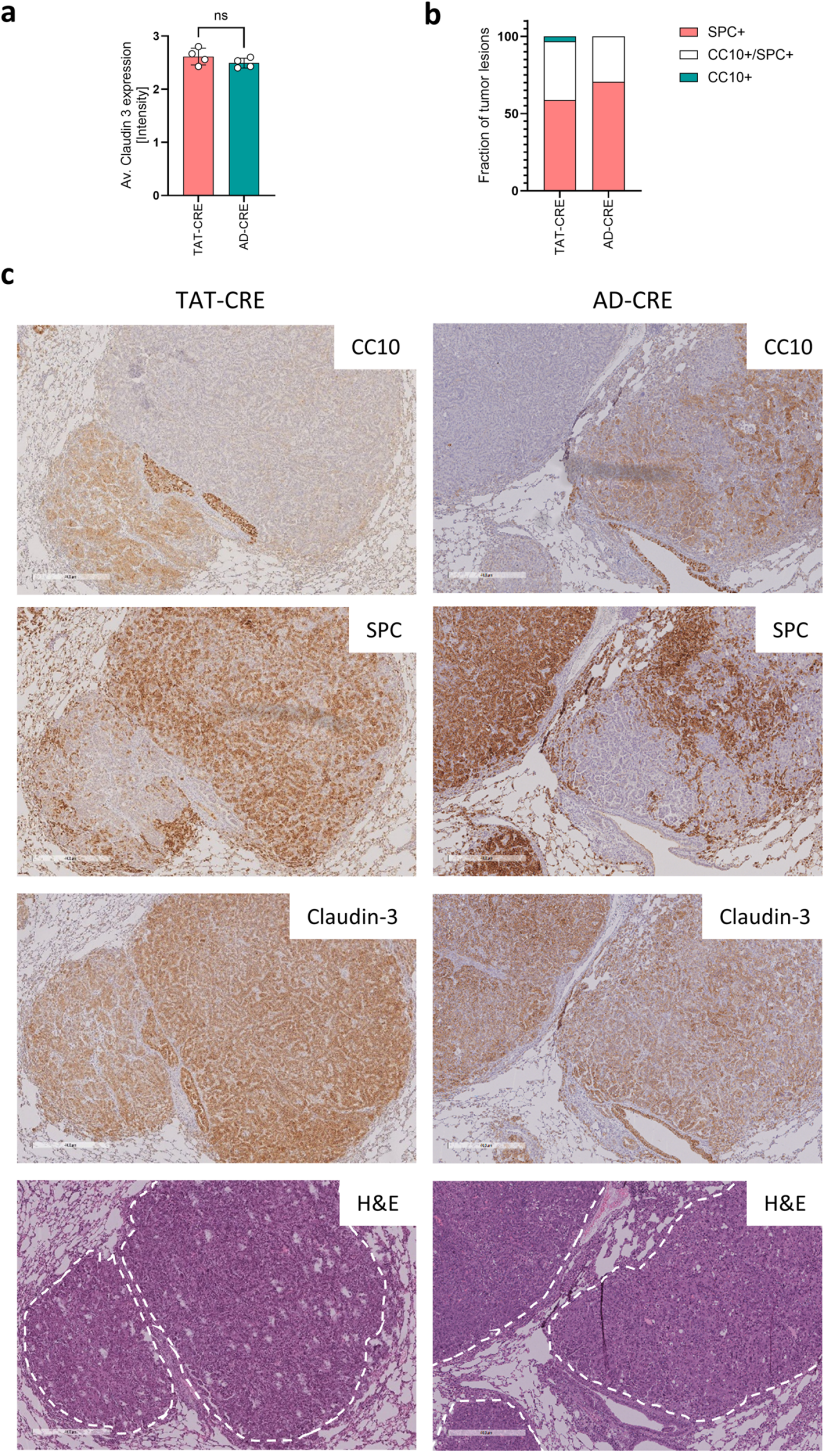
TAT-CRE induced lung tumors expressed CC10 more frequently than AD-CRE induced lung tumors. Lung adenocarcinomas induced by TAT-CRE (*n* = 4, 2 males, 2 females) and AD-CRE (*n* = 4, 2 males, 2 females) inhalation were analyzed for pulmonary markers by IHC. **a** The average staining intensity of Claudin-3 was calculated taking into account the average of all tumor lesions per slice. **b** Tumor lesions were classified as SPC+; CC10+/SPC+ and CC10+, respectively. A tumor lesion was defined by a minimal diameter of 100 µm. Tumor lesions are indicated in the H&E stain by a white dashed line. p-values were calculated using an unpaired two-sided t-test. Data is shown as mean ± SD. P-values ≤ 0.05 are considered as significant. **c** Representative IHC stain of CC10, SPC, Claudin-3 and H&E, magnification 6x. Bars indicate 400 µm.

For the reason that ATII cells, Club cells and BASCs can serve as tumor cell-of-origin^12,26^ we assessed the expression of the markers CC10, SPC and claudin-3, as a general marker of the pulmonary epithelium^27^. Lung adenocarcinomas developing after AD-CRE and TAT-CRE application expressed claudin-3 similarly (Fig. 2a). AD-CRE and TAT-CRE induced tumors showed predominantly SPC expression, but upon TAT-CRE lung tumor induction substantially more CC10 positively stained lesions occur (Fig. 2b). Representative IHC stains showed that CC10-positive and SPC-positive tumors can arise in the same lung (Supplemental Fig. 1) and in close proximity (Fig. 2c).

**Fig. 3:**
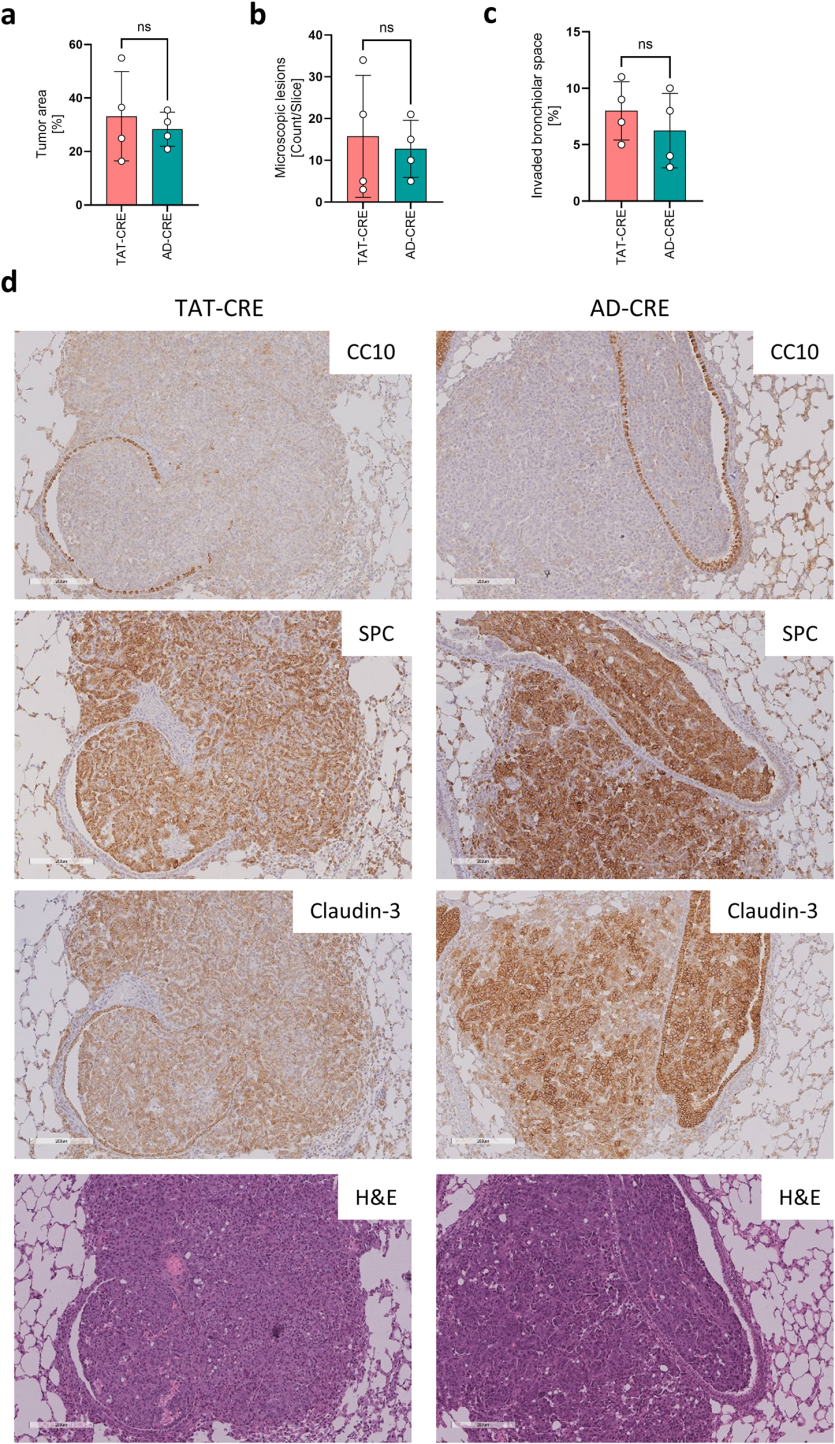
Lung cancer induced by TAT-CRE and AD-CRE had a comparable capacity to invade the bronchiolar space. Lung adenocarcinomas induced by TAT-CRE (n = 4, 2 males, 2 females) and AD-CRE (n = 4, 2 males, 2 females) inhalation were analyzed regarding bronchiolar invasion based on IHC stains. **a**, **b** Tumor area and microscopic lesions was determined based on Claudin-3 positive tumor lesions normalized to total lung area per slice. A tumor lesion was defined by a minimal diameter of 100 µm. **c** Invaded bronchiolar space was determined based on the evaluation of CC10 and SPC IHC stains, taking into account the average of all detectable bronchiolar spaces per slice. **d** Representative IHC stains of CC10, SPC, Claudin-3 and H&E, magnification 10x. Bars indicate 200 µm. p-values were calculated using an unpaired two-sided t-test. Data is shown as mean ± SD. P-values ≤ 0.05 are considered as significant.

In a next step, we analyzed the growth pattern of microscopic lung adenocarcinomas. The tumor area (Fig. 3a), the count of microscopic lesions (Fig. 3b) and invaded bronchiolar spaces (Fig. 3c) were determined based on CC10, SPC, and claudin-3 IHC stains, respectively (Fig. 3d). We did not detect considerable differences in these three measures comparing TAT-CRE and AD-CRE induced lung adenocarcinomas.

**Fig. 4:**
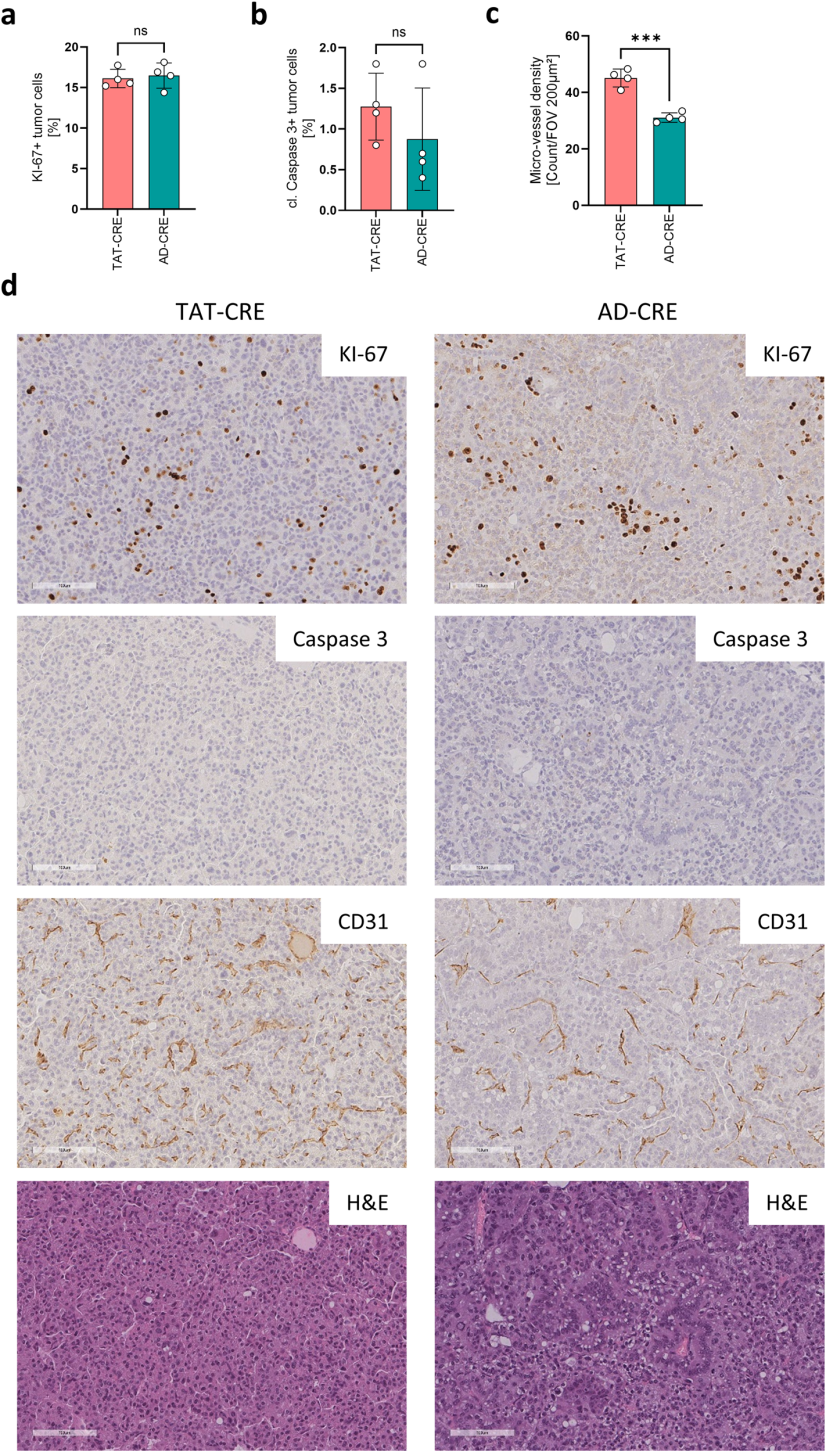
TAT-CRE induced lung tumors showed significantly increased micro-vessels compared to AD-CRE induced lung tumors. Lung adenocarcinomas induced by TAT-CRE (n = 4, 2 males, 2 females) and AD-CRE (n = 4, 2 males, 2 females) inhalation were analyzed regarding cell proliferation, micro-vessel density and apoptosis based on IHC stains. **a** KI-67 stain was analyzed to determine the cell proliferation rate, taking into account the average of all tumor lesions per slice. **b** Cleaved Caspase 3 stain was analyzed to determine the apoptosis rate, taking into account the average of all tumor lesions per slice. **c** CD31 stain was analyzed to determine the micro-vessel density per field of view (FOV) of 200 µm². **d** Representative IHC stains of KI-67, CD31, cleaved Caspase-3 and H&E, magnification 20x. Bars indicate 100 µm. p-values were calculated using an unpaired two-sided t-test. Data is shown as mean ± SD. P-values ≤ 0.05 are considered as significant.

To get an impression of tumor cell proliferation and apoptosis rates, we analyzed KI-67 and cleaved Caspase-3, respectively (Fig. 4a, b). TAT-CRE and AD-CRE induced lung adenocarcinomas showed similar proliferation rates of around 16% and similar apoptosis rates of around 1%.

In summary, macroscopic and microscopic evaluation of lung adenocarcinomas using µCT and IHC revealed corresponding tumor induction and tumor growth characteristics of TAT-CRE and AD-CRE induced lung adenocarcinomas.

### TAT-CRE induced lung tumors differ from AD-CRE induced lung tumors in the abundance of tumor vessels and Club cells

As angiogenesis is a central feature in tumor growth^28^, we investigated micro-vessel density by assessing CD31 positive vessels in TAT-CRE and AD-CRE induced lung adenocarcinomas. Interestingly, TAT-CRE induced lung tumors harbored significantly enriched micro-vessels (p = 0.0002; 95% CI −18.43 to −9.635) compared to AD-CRE induced lung tumors (Fig. 4c, d).

**Fig. 5:**
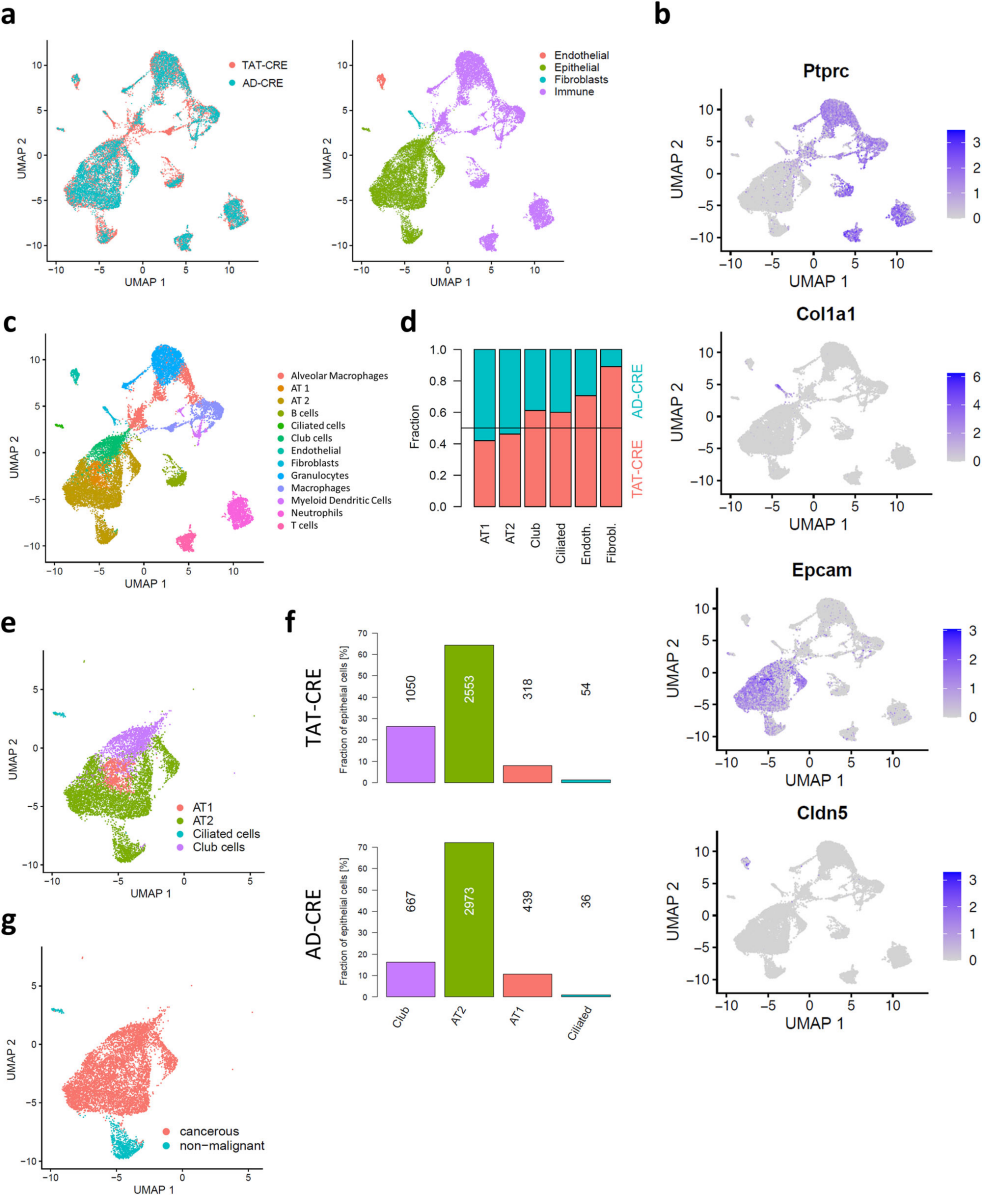
TAT-CRE induced lung tumors harbored a considerable higher fraction of endothelial cells and fibroblasts than AD-CRE induced lung tumors. **a** Uniform manifold approximation and projection (UMAP) representation of the integrated scRNA-seq data of TAT-CRE and AD-CRE induced lung adenocarcinoma samples (n = 1 per group, 2 males), colored by dataset (left) and by cell category (right). Each dot represents a single cell. **b** UMAP indicating lineage marker expression of Ptprc (immune), Epcam (epithelial), Col1a1 (fibroblasts) and Cldn5 (endothelial). **c** UMAP visualization of the integrated scRNA-seq data of TAT-CRE and AD-CRE induced lung adenocarcinoma samples, colored by inferred cell type. **d** Stacked bar plot showing the fraction per condition (TAT-CRE vs AD-CRE) among non-immune cell types: AT1 (alveolar type 1) cells, AT2 (alveolar type 2) cells, Club cells, ciliated cells, endothelial cells and fibroblasts. **e** UMAP representation of the integrated scRNA-seq data of epithelial cells only, colored by cell type. **f** Relative amount of epithelial cell types among epithelial cells identified for TAT-CRE and AD-CRE induced lung adenocarcinoma samples, respectively, with numbers indicating the corresponding absolute cell numbers. **g** UMAP representation of the integrated scRNA-seq data of epithelial cells only, colored by malignancy.

We hypothesized that there might be differences between TAT-CRE and AD-CRE induced lung adenocarcinomas in the TME. Thus, we performed scRNA-seq of one TAT-CRE and one AD-CRE induced lung tumor. The analyzed lesion was in both cases located in the lung periphery and represented the target lesion referring to the biggest macroscopic lesion in the lung upon Cre-recombination. The different cell lineages in the TME were subcategorized using the immune marker *Ptprc* encoding CD45, the fibroblast marker *Col1a1* encoding type-I collagen, the epithelial marker *Epcam* encoding CD326 and the endothelial marker *Cldn5* encoding claudin-5 (Fig. 5a, b). Different cell types are clustered, integrated and visualized by UMAP. Immune cell types comprised among others macrophages and specifically alveolar macrophages (Fig. 5c). Of note, also this scRNA-seq data set indicated a higher fraction of endothelial cells in the TAT-CRE induced lung tumors compared to AD-CRE lung tumors. Another cell fraction which was substantially more abundant in TAT-CRE induced lesions was made up of fibroblasts (Fig. 5d). The epithelial cell types indicated few ciliated cells and ATI cells and predominantly Club cells and ATII cells upon TAT-CRE and AD-CRE induction. Interestingly, scRNA-seq indicated an increased ratio of Club cells to ATII cells upon TAT-CRE vs AD-CRE lung adenocarcinomas, with 0.41 and 0.22, respectively (Fig. 5e–g).

We assessed malignant transformation of epithelial cells upon TAT-CRE and AD-CRE. Ciliated cells were not transformed upon TAT-CRE and AD-CRE but were more abundant in the TAT-CRE sample. Club cells appeared to be transformed to a higher extend in the TAT-CRE than the AD-CRE condition. AD-CRE induced malignant transformation in a higher fraction of ATII cells. ATI cells showed similar and full transformation upon TAT-CRE and AD-CRE (Supplementary Fig. 2a). In addition, tumor regions induced upon TAT-CRE and AD-CRE represented an increase in phospho-ERK1/2 indicating Kras pathway activation compared to normal adjacent tissues (Supplementary Fig. 2b). Finally, analyzing all malignant epithelial cells vs non-malignant epithelial cells, we found transcripts of the Kras- and p53-target *Atf3*^29,30^ to be significantly enriched in cancerous cells (p = 0.0072; 95% CI −0.1302 to −0.02038) (Supplementary Fig. 2c). Next, we used *Arf3* transcripts as a surrogate for potential recombination of stromal cell types including fibroblasts, endothelial cells and macrophages. Only macrophages showed increased transcripts of *Arf3* upon TAT-CRE vs AD-CRE (p = 0.0388; 95% CI 0.005315 to 0.2001) (Supplementary Fig. 2d).

Consequently, scRNA-seq data strengthen the findings obtained from IHC stain evaluation, that TAT-CRE induced lung tumors showed considerable differences in tumor vessels and tumor cell-of origin.

### TAT-CRE induced lung adenocarcinomas harbored an altered TME regarding tumor-associated macrophages

**Fig. 6:**
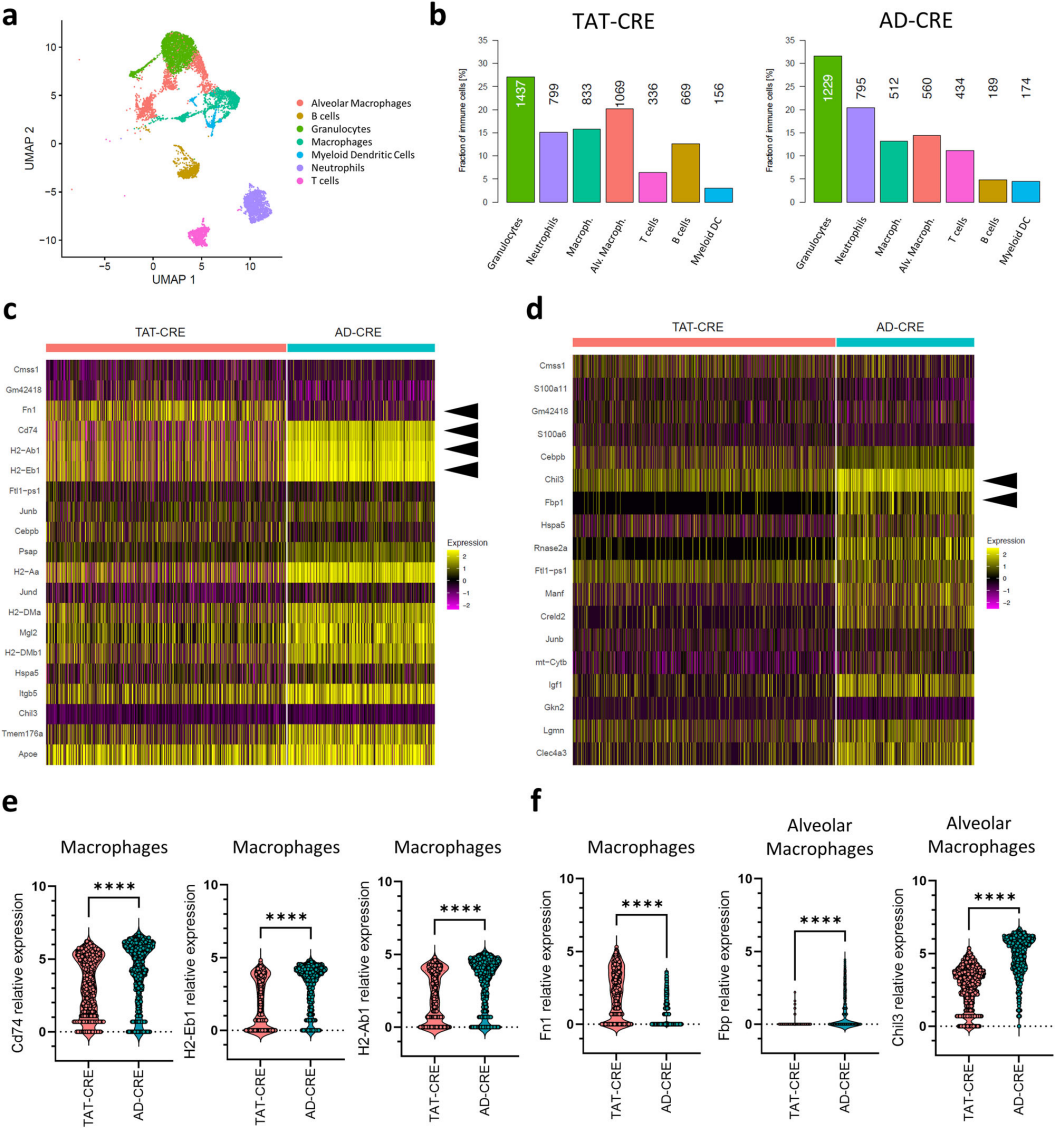
Pro-tumorigenic macrophages were more abundant in TAT-CRE vs AD-CRE induced lung tumors. **a** UMAP representation of the integrated scRNA-seq data of immune cells only for TAT-CRE and AD-CRE induced lung adenocarcinoma samples (n = 1 per group, 2 males), colored by identified cell types. Each dot represents a single cell. **b** Relative amount of immune cell types among immune cells identified for TAT-CRE and AD-CRE induced lung adenocarcinoma samples, respectively, with numbers indicating the corresponding absolute cell numbers. **c**, **d** Heatmap showing the top 20 differentially expressed genes (DEGs) in macrophages and alveolar macrophages, respectively. Arrows indicate MHC-II-related and angiogenesis-related genes. **e** Differentially expressed MHC-II-related genes in macrophages of TAT-CRE and AD-CRE induced lung adenocarcinoma samples. **f** Differentially expressed angiogenesis-related genes in macrophages and alveolar macrophages of TAT-CRE and AD-CRE induced lung adenocarcinoma samples. p-values were calculated using an unpaired two-sided t-test. Data is shown as mean ± SD. P-values ≤ 0.05 are considered as significant.

Based on the differential abundance of tumor micro-vessels, it is likely that TAT-CRE and AD-CRE induced lung adenocarcinomas harbored a different TME composition of immune cells. We investigated immune cell types pre-classified by *Ptprc* and visualized them using a UMAP. The following immune cell types have been identified: granulocytes, neutrophils, macrophages, specific alveolar macrophages, T cells, B cells and myeloid dendritic cells (DC) (Fig. 6a).

Macrophages have been previously described as mediators of tumor angiogenesis^31,32^ and showed increased frequencies in TAT-CRE compared to AD-CRE induced lung tumors (Fig. 6b). We assessed the differentially expressed genes in macrophages and alveolar macrophages (Fig. 6c, d). AD-CRE induced lung tumors harbored macrophages with significantly increased expression of MHC-II related genes: *Cd74 (p* < *0.0001; 95% CI 0.8005 to 1.183)*, *H2-Ab1* (p < 0.0001; 95% CI0.9057 to 1.226) and *H2-Eb1* (p < 0.0001; 95% CI 1.034 to 1.338) (Fig. 6e). The pro-angiogenic factor *Fn1*^33^, encoding for cellular fibronectin, was significantly increased in macrophages of TAT-CRE induced lung tumors (p < 0.0001; 95% CI −1.745 to −1.501) (Fig. 6f). Alveolar macrophages of TAT-CRE induced lung tumors showed decreased anti-angiogenic markers *Fbp*^34^ encoding fructose-1,6-bisphosphatase (p < 0.0001; 95% CI 0.7042 to 0.8499) and *Chil3*^31^ encoding Ym1 (p < 0.0001; 95% CI 1.963-2.239). Consequently, macrophages and alveolar macrophages showed a pro-angiogenic expression profile (Fig. 6f).

**Fig. 7:**
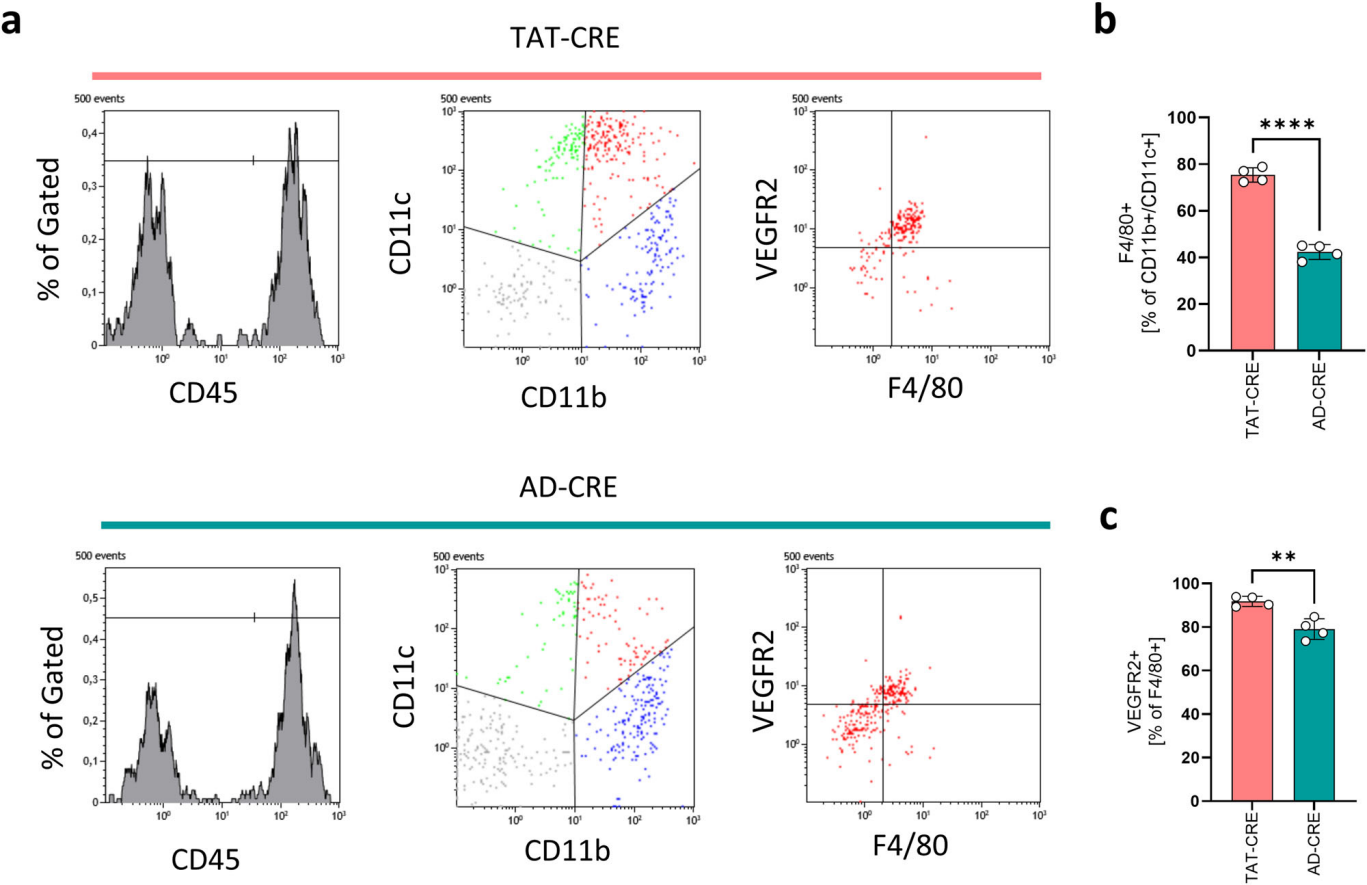
TAT-CRE induced lung tumors harbored an increased fraction of VEGFR2 positive macrophages compared to AD-CRE induced lung tumors. Lung adenocarcinomas induced by TAT-CRE (n = 4, 2 males, 2 females) and AD-CRE (n = 4, 2 males, 2 females) inhalation were analyzed by flow cytometry. **a** Representative histograms, dot plots and the gating of CD45, CD11c, CD11b, F4/80 and VEGFR2 stains. **b** F4/80 was analyzed to determine the macrophage rate in the CD11+/CD11b+ immune cell population. **c** VEGFR2 was analyzed as a pro-angiogenic marker on macrophages. p-values were calculated using an unpaired two sided t-test. Data is shown as mean ± SD. P-values ≤ 0.05 are considered as significant.

Analyzing the pro-angiogenic marker VEGFR2^35^ on tumor associated macrophages by flow-cytometry revealed that macrophages are significantly enriched (p < 0.0001; 95% CI −38.53 to −27.59) in the tumor proximity of TAT-CRE induced lung adenocarcinomas (Fig. 7a, b). Moreover, tumor-associated macrophages more frequently express VEGFR2 (p = 0.0029; 95% CI −19.21 to −6.286) (Fig. 7c), supporting the findings obtained from scRNA-seq.

In order to assess a possible Cre-recombination in macrophages, we used a Cre-reporter mouse with a luciferase expression cassette and treated isolated macrophages from the lung in vitro with Cre-recombinase. 24 h after induction a robust bioluminescence was detected (p < 0.0001; 95% CI 1739 to 1991, and 473.5 to 886.5, respectively) (Supplementary Fig. 3a, b). Next, we isolated pulmonary macrophages from the *B6.129-Kras*^*tm4Tyj*^ *Trp53*^*tm1Brn*^*/J* mouse model and checked transcripts of the secreted pro-angiogenic key factor *Vegfa* upon Cre-based induction. Cre-recombination, used to activate mutated *Kras* and deplete *Trp53*, was able to increase expression of *Vegfa* in macrophages in vitro (p = 0.0397; 95% CI 0.04807 to 1.205) (Supplementary Fig. 3c). Finally, taking the scRNA sequencing data of TAT-CRE and AD-CRE induced lung tumors into account, significantly increased transcripts of *Vegfa* were found in macrophages upon TAT-CRE vs AD-CRE (p < 0.0001; 95% CI −0.2273 to −0.1415) suggesting an increase in *Vegfa* upon CRE-based activation of mutated *Kras* and depletion of *Trp53* also in vivo (Supplementary Fig. 3d).

Taken together, TAT-CRE induced lung cancer showed not only differential abundance of micro-vessels but also an increased pro-angiogenic signature on tumor-associated macrophages compared to AD-CRE induced lung cancer. These results indicated that TAT-CRE induces a more angiogenic and immune-reactive TME than AD-CRE. Nevertheless, these seemingly pro-tumorigenic aspects do not significantly alter the development and growth of lung tumors after TAT-CRE induction.

## Discussion

The frequently used Cre-recombinase induction systems based on cross-breeding Cre-strains or applying adenovirally expressed Cre-recombinase raise two challenges: addressing the desired reduction of animal numbers and accessibility of infrastructures and facilities with the required safety level, respectively. For this reason, we comprehensively compared GE-biosafety level S1 TAT-CRE and S2 adenoviral Cre-recombinase (AD-CRE) to induce lung adenocarcinomas driven by a Kras^G12D^ mutation and functional p53 depletion^19,21^.

First, we elucidated common features regarding tumor growth characteristics based on tumor induction and progression, non-target lesions, survival and bronchiolar invasion. Moreover, tumor cell proliferation and apoptosis rates showed similar levels comparing TAT-CRE and adenoviral Cre-recombinase induced lung tumors. Our findings clearly demonstrate that TAT-CRE can be successfully used to induce site-specific tumors at GE-biosafety level S1 comparable to adenoviral Cre-recombinase. This may lower boundaries regarding the access to resources and facilities with GE-biosafety level S2 and may improve research using Cre-inducible cancer models with accessible inoculation sites such as sarcomas with intramuscular or subcutaneous injection^16^, head and neck carcinomas with application at the tongue^36,37^, and breast cancer^38^ by lowering animal numbers.

Second, we observed ATII cells as the major tumor cells-of-origin in TAT-CRE and AD-CRE induced lung carcinomas based on IHC and scRNA-seq data, although the fraction of malignant Club cells expressing the marker CC10 was significantly higher in TAT-CRE induced lung tumors. Corresponding to our data, the pulmonary cell types Club Cells (CC10+), ATII cells (SPC+) and BASCs (CC10+SPC+) are described as the three sources of lung adenocarcinomas^12,26^. Moreover, we showed that CC10 positive and SPC positive tumors can arise in close proximity in the same lung which is in line with previous publications^39^. In addition, TAT-CRE and AD-CRE give rise to cancerous ATI cells, which was recently discovered by using *Gramd2*-CreERT2 mice crossed with a conditional *Kras*^*G12D*^ expressing mouse strain^40^.

Third, we highlighted considerable differences in tumor vessels and macrophage populations between TAT-CRE and AD-CRE induced *Kras*^*G12D*^-driven lung adenocarcinomas. Thereby, the macrophages showed different levels of pro-angiogenic markers suggesting that the macrophages might dictate the differential vessel abundance in TAT-CRE and AD-CRE induced lung adenocarcinomas. This was strengthened by our in vitro recombination experiments using pulmonary macrophages of the *B6.129-Kras*^*tm4Tyj*^ *Trp53*^*tm1Brn*^*/J* showing an increase in *Vegfa* transcripts upon Cre-recombination. In lung adenocarcinoma patients, the expression of angiogenic proteins VEGF-A, VEGFR1 and VEGFR2 is significantly associated with *KRAS* mutations^41^ and possible therapy options comprise anti-angiogenic agents^42^.

It was described that adenoviral Cre-recombinase used to induce lung cancer also transduced macrophages^43^. TAT-CRE induced lung tumors showed enriched micro-vessel density and pro-angiogenic macrophage populations, indicated by high Fn1^33^, low Fbp^34^, low Chil3^31^ and VEGFR2 expression^35^. This may indicate that TAT-CRE and AD-CRE provide different levels of transduction efficacy in macrophages. However, our in vitro recombination experiments using pulmonary macrophages of a Cre-reporter mouse showed that AD-CRE seems to be more potent than TAT-CRE to induce recombination in similar concentration as applied in vivo. The discrepancy to the in vivo finding might by caused by higher immune mediated elimination of the viral vector. Neutrophils, which were more abundant in the AD-CRE setting in vivo (Fig. 6b), are known to interact with adenoviral vectors via Fc receptors leading to an uptake of the vector^44^.

In both cases, off-target effects on macrophages caused by direct Cre-recombinase application may alter the tumor microenvironment in the used model compared to naturally occurring lung cancer. Nevertheless, our data indicate that the potential differential intensity of the off-target effects on macrophages does not significantly alter lung adenocarcinoma induction and tumor progression. A reason for almost identical tumor growth upon TAT-CRE and AD-CRE induction might be that mutant *Kras*-driven cancer cells evade innate immune surveillance by hindering macrophage phagocytosis^45^. It has been recently reported in lung adenocarcinoma patients and a tumor cell injection model that activated KRAS signaling triggers CD47 expression on tumor cells inhibiting their phagocytotic elimination^45^.

Further studies should determine if the macrophage off-target effect affects therapy responses in preclinical cancer models since this could add another level of complexity to interpret treatment effects. Particularly, the efficacy of immune checkpoint blockade might be influenced by recombination in macrophages, as macrophage phagocytosis is enhanced under anti-PD-1 treatment^46^. Moreover, future research could investigate whether the pro-angiogenic TME induced by TAT-CRE affects the response to anti-angiogenic drugs.

Off-target effects may be reduced in the future, by refining delivery mechanisms of Cre-recombinase for example by using nanoparticles liberating their content at specific body sites by contact to target-molecules. Gesicles filled with Cre-recombinase are a first step in this direction^47^. In the field of lung cancer, driving Cre-recombinase expression under the control of specific promotors such as SFTPC for targeting ATII cells, SCGB1A1 for targeting Club cells, and calcitonin gene-related peptide (CGRP) to target neuroendocrine pulmonary cells, will likely minimize the off-target effect on the non-epithelial cell compartments in the lung. However, this is currently only achieved by using adenoviral particles^4^ requiring a biosafety level S2 infrastructure.

In summary, we postulate that the usage of GE-biosafety level S1 TAT-CRE can be easily implemented in other Cre-inducible cancer models ex vivo and in vivo with accessible inoculation sites. This means that boundaries in cancer research due to limited access to S2 resources and facilities can be lowered by implementing TAT-CRE for tumor induction and opens another door to methodologies such as mini-experiment design and Bayesian updating^8^ to reduce animal numbers.

Taken together, TAT-CRE represents a valuable alternative to adenoviral Cre-recombinase and may democratize cancer research, enabling more laboratories to conduct advanced studies without S2-level restrictions.

## Methods

### In vivo experiments

We adhere to the ARRIVE guidelines 2.0 for reporting animal research^48^. The genetically engineered mouse model *B6.129-Kras*^*tm4Tyj*^ *Trp53*^*tm1Brn*^*/J* (JAX stock #032435) was used to induce lung adenocarcinoma driven by a Cre-inducible conditional Kras^G12D^ mutant and functional Trp53 knock out, as previously described^19,21^ and was kindly provided by H. C. Reinhardt (Department of Hematology and Stem Cell Transplantation, University Hospital Essen, Germany). Male and female C57BL/6 J mice with at least 20 g body weight were anesthetized with Ketamin/Xylazin (100 mg/kg/BW (body weight) i.p./0.5 mg/kg/BW i.p.) for nasal inhalation of TAT-CRE and AD-CRE diluted in PBS. The mouse is held in the hand so that its ventral side is facing upwards. The mouse is then tilted so that its head is above its feet. The pipette tip is placed directly over the opening of a nostril and the solution is slowly administered in a dropwise manner with a maximum volume of 62.5 µL^49^. AD-CRE (Ad5CMVCre) was provided by the University of Iowa Viral Vector Core and applied with a concentration of 2.0*10^7 pfu, which we have previously shown to be potent to induce lung carcinomas driven by mutated *Kras*^13,50^. TAT-CRE (Cat.-No. SCR508, Merck Millipore) was applied with a concentration of 100 units corresponding to ~2 µM, which we have previously successfully used in vitro to induce recombination in murine cells^50^. Serial µCT measurements were performed to monitor tumor induction, tumor growth and non-target-lesions. Mice were randomized to the TAT-CRE and the AD-CRE cohort before tumor induction but Cre-recombinase was applied in a not-blinded manner. 3 weeks after virus inhalation, once a week, mice were anesthetized with isoflurane to perform a µCT scan (LaTheta mCT, Hitachi Alcoa Medical, Ltd).

We measured the regions of interest using the pre-installed ‘Volume’ protocol with slow speed (18 sec rotation time) and ‘high’ X-ray voltage referring to 50 kV with a constant current of 1 mA. The micro-focus x-ray tube has a focal spot size of 50 µm^51^. The mice were posted face down and head front. Artefact removal was set to pre-installed ‘Soft tissue and Lung’. The detector consists of a photodiode array with 512 pixels of 0.45 × 0.6 mm^51^. CT images are displayed at a scale of −300 to 300 with radius pixels set to 1.0 in monochrome format. Prior to CT scans, the system calibrated air value, central channel, BMD stability and standard phantom using the phantom PHA-201 for mice (Aloka Co., Ltd., Tokyo, Japan) with a cross-section size of 2.5 cm and length of 10 cm manufactured from polymethyl methacrylate^51^. The mouse phantom revealed an average BMD of 1433.2 mg/cm³ for low energy and 1442.4 mg/cm³ for high energy. The size of the trans-axial field-of-view was set to ‘L’.

Tumor progression was measured by mouse adapted RECIST criteria v1.1, as published previously^52^ with an adapted slice thickness of 0.3 mm. A maximal tumor size of 10 mm in diameter was not exceeded. DICOM files were analyzed using OsiriX (aycan Digitalsysteme GmbH). Probability of survival was analyzed as Kaplan-Meier-Curves and statistically evaluated using Mantel-Cox test (GraphPad Prism). Survival analysis was performed latest until the human end-point was reached. The human end-point was defined by a score of = 20 according the approved score sheet to monitor health and suffering on a daily basis. The human end-point was specified by a reduction in weight ≥ 20% compared to the time point of first tumor detection, or by markedly increased abdominal breathing to prevent the occurrence of dyspnea indicated by pumping breathing with strong involvement of the abdominal muscles. Body weight was determined using a laboratory precision balance with a weighing capacity of 500 g and accuracy of 0.1 g on the basis of scoring, which is part of the regular determination of health and suffering of experimental animals in Germany. Lung weight was determined directly after harvest of the lungs using a similar laboratory precision balance.

### Immunohistochemistry

Lungs of mice were harvested at a progressed tumor stage at maximum of 14 weeks after inhalation when the tumor volume substantially increased over time and reached a tumor volume of >100 mm³. Mice were sacrificed by cervical dislocation, the peritoneum and the rib cage were opened to remove the whole lung. Tissue was harvested, and paraffin embedded. 3 µm tissue sections were deparaffinized and immunohistochemically stained. All sections receive hematoxylin & eosine (H&E) stain. In brief, Hematoxylin Solution Gill No. 3 (6 g/L) by Sigma- Aldrich and Eosin Y Solution (0,5%) by Sigma- Aldrich were used. Proteins of interest were stained using primary antibodies, the secondary biotinylated goat anti-rabbit antibody (Biozol) or biotinylated horse anti-mouse (Biozol) and the detection kit (Vecatstain Elite ABC-HRP Kit, Biozol). Primary Antibodies used: KI-67 (D3B5, Cell Signaling, #12202, 1:2000, TRS6 1 minute), cleaved Caspase 3 (polyclonal, Cell Signaling, #9661, 1:400, TRS6 1 minute), CD31 (7O7K3, Thermo Fischer Scientific, MA5-37858, 1:400, TRS6 1 minute, AB-Block), uteroglobin (also known as CC10, polyclonal, Thermo Fischer Scientific, PA5-102469, 1:20000, TRS6 10 minutes, AB-Block), surfactant protein C (SPC, polyclonal, Thermo Fischer Scientific, PA5-102493, 1:1000, Citrate 1 minute), claudin-3 (polyclonal, Thermo Fischer Scientific, # 34-1700, 1:500, Citrate 1 minute), phospho-p44/42 MAPK (Erk1/2) (Thr202/Tyr204) (20G11, Cell Signaling, #4376, 1:1000, TRS6 1 minute). The antibody validation occurred as follows. CD31 and claudin 3 antibodies have been validated by the distributor using genomic knock-out. CC10, SPC, and KI-67 have been validated by the distributor on control tissue, testis, lungs and colon, respectively. Cleaved caspase-3 was validated by the distributor using Cytochrome C stimulation of cancer cells. We have validated all antibodies for specific binding pattern and optimal concentration on murine lung tissue and if applicable, also on human lung tissue. Different antibody concentrations have been tested for each antibody and the most descriptive one was chosen.

For Epitope retrieval, a method of cull, each antibody was tested with TRS6 (Cat.-No. S1699, Dako) and self-made citrate buffer (10.5 g citric acid monohydrate added to 5 L aqua dest., followed by titration with sodium hydroxide to pH 6.0). TRS6 is a commercially available citrate buffer system containing additional preservatives and stabilizers. Compared to self-made citrate buffer, TRS6 is easier in handling but more cost extensive. Both buffer sytems were tested and the sytem resulting in lowest background staining was chosen.

Staining was performed as follows: The deparaffinized slices were treated with 3% H_2_O_2_ for 10 minutes and heat induced epitope retrieval performed in a pressure cooker in citrate buffer or TRS6 at 120 degrees for 1 – 10 minutes depending on the antibody. After cooling down for 30 minutes and optional AB- Blocking for 10 minutes the slices were incubated with the primary antibody overnight at room temperature. The detection was performed by the secondary antibody followed by the avidin-biotin complex and 3,3′-Diaminobenzidine (DAB) as the chromogen. Finally, the slices were counterstained with Mayer’s hemalum solution (Merck Millipore). Slices were scanned by the S210-Scanner (Hamamatsu TV Co., Ltd.) and analyzed using Aperio ImageScope (Leica Biosystems) and ImageJ (National Institute of Health, US) in a blinded manner.

A tumor lesion on a slice was classified by a minimal diameter of 100 µm. The average claudin-3 expression was determined per mouse by two independent examiners. To determine the average by stain intensity, all detectable tumor lesions per slice were analyzed and categorized with 0-3 (no, weak, moderate or high stain intensity). The localization of claudin-3 was not taken into account.

To determine the fraction of SPC+; CC10+/SPC+ and CC10+ tumor lesions, all detectable tumor lesions per slice were analyzed using ImageJ. First, DAB brown stain was separated from the blue counterstain using the Color Deconvolution2 plugin which appears under the Image>Color tab. In the Color Deconvolution pop-up window, H DAB option was selected from the Vectors menu. In order to specify the measurements to be displayed, Set Measurement option was chosen from the Analyze tab. The Integrated Density box was selected in the pop-up window. Next, the Color 2 window which indicates DAB staining, was selected. To set a threshold for the DAB stain, the Threshold option was selected under the Image>Adjust tab. The minimum and maximum threshold values were adjusted to measure the positively stained area within a tumor lesion. Each tumor lesion was defined by selecting the freehand tool and specifying the region of interest. Finally, the presence of CC10+ and SPC+ areas per tumor lesion were determined using the measure option which is found under the Analyze tab.

The micro-vessel density was determined using ImageJ by counting the micro-vessels in a field of view (FOV) of 200 µm². The images were analyzed as described in details above. The minimum threshold value was set to 100 and the maximum value was set to 200. Then, the FOV was defined by selecting the rectangle tool and specifying the region of interest. Finally, the micro-vessel density was determined by counting stained areas per FOV using the measure option which is found under the Analyze tab.

### Flow Cytometry

Lungs of mice were harvested at a progressed tumor stage at maximum of 14 weeks after inhalation when the tumor volume substantially increased over time and reached a tumor volume of > 100 mm³. Mice were sacrificed by cervical dislocation, the peritoneum and the rib cage were opened to remove the whole lung. Cells were isolated from the lungs using 40 µm cell strainers (BD Falcon) after mechanical dissociation. ACK lysis buffer (Life Technologies) was applied 10 min at room temperature to lyse red blood cells. Cells were washed with PBS prior to staining, epitope retrieval as a method of cull has not been performed. Cell suspensions are stained for 30 min at 4 °C using primary antibodies and isotype controls, both obtained from BioLegend if not otherwise specified: CD45 (FITC, 30-F11), CD11c (PE-Dazzle594, N418), CD11b (PE-Cy7, M1/70), F4/80 (Alexa Fluor 700, BM8), VEGFR2 (PerCP-Cy5.5, 89B3A5), Rat IgG2aΚ (PerCP-Cy5.5, Alexa Fluor 700), PE-Dazzle594 Armenian Hamster IgG (PE-Dazzle594), Rat IgG2bΚ (FITC, PE-Cy7). Cell viability was assessed by staining with Zombie Aqua Fixable Viability Kit (BioLegend). CD45 was used as a marker for immune cells and CD11b was used to identify immune cells of the myeloid lineage. CD11c was used as a marker for dendritic cells and alveolar macrophages, and in a next step F4/80 positivity was needed to discriminate macrophages from other myeloid cells such as eosinophils. Flow cytometry measurements were performed on a Gallios 10/3 (Beckman Coulter) and data was analyzed using Kaluza in a blinded manner (Beckman Coulter).

### Macrophage stimulation

Pulmonary macrophages were isolated from the *B6.129-Kras*^*tm4Tyj*^ *Trp53*^*tm1Brn*^*/J* mouse (JAX stock #032435) and a Cre-reporter mouse *Gt(ROSA)26Sor*^*tm1(Luc)Kael*^*/J* (JAX stock #034320)^53^ where the luciferase gene is blocked by a *loxP*-flanked STOP fragment placed between the *luc* sequence and the *Rosa26* promotor, respectively. Mice were sacrificed by cervical dislocation, the peritoneum and the rib cage were opened to remove the whole lung. After mechanical dissociation of the lung, 40 µm cell strainers (BD Falcon) were used to achieve single cells. ACK lysis buffer (Life Technologies) was applied 10 min at room temperature to lyse red blood cells. Cells were washed with PBS prior to magnetic activated cell sorting using the Monocyte Isolation Kit (130-100-629, Miltenyi Biotech) according to manufacturer’s advice. 20,000 macrophages were seeded in 100 µL RPMI medium in 96-wells and incubated with 100 U/ml TAT-CRE and 2*10^7 pfu/ml AD-CRE for 20 h. Luciferase activity was analyzed using ONE-Glo™ Luciferase Assay System (Promega) according to manufacturer’s advices. Prostaglandin E2 (PGE2) (0.5 ng/ml) for 20 h was used as a positive control to stimulate macrophages to upregulate angiogenic markers^54^. For RNA isolation from macrophages, 200.000 pulmonary macrophages were treated as described and RNA was isolated using the RNeasy Mini Kit (Qiagen) according to manufacturer’s advices.

### Real-Time PCR

RNA concentration was measured by NanoDrop and 500 ng were used for reversed transcription with the RevertAid First Strand cDNA-Synthesis-Kit (Thermo Scientific) according to manufacturer’s advices. Quantitative PCR for targets was performed using Fast Start SYBR Green (Merck) and the primer sequences as follows (5′-3′): *Vegfa*-fwd AGCAGAAGTCCCATGAAGTGA; *Vegfa*-rev ATGTCCACCAGGGTCTCAAT^54^. *18S*-fwd ACAGCCAGGTTCTGGCCAACGG; *18S*-rev TGACCGCGGACAGAAGGCCC. To determine the relative expression level of transcripts, Gapdh was used for normalization followed by the ΔΔCT-method. Real-Time PCR was performed in a blinded manner.

### scRNA-seq

Single-cell RNA sequencing of lung tumors was performed by the Singleron Biotechnologies GmbH in Cologne, Germany. A piece of the tumor tissue with a diameter of 3-5 mm was embedded in Sample Preparation Buffer (Singleron Biotechnologies) stable for up to 72 hours prior processing. Single cell suspension from tumor tissue were processed in sCelLiVETM Tissue Dissociation Buffer. Then, single cells were loaded into the microfluidic SCOPE-chipTM and cell identifying tags were flowed in and allowed to settle in the wells on top of the cells. mRNA from each lysed cell was hybridized to the barcode sequences on the bead in the same well. Then, reverse transcription of primed RNA and cDNA amplification take place. The amplified cDNA was fragmented, adapter ligated and amplified to construct a sequencing library suitable for the Illumina sequencing platform.

The analysis of the scRNA-seq data was performed within R version 4.3.2 using Seurat-package 5.0.1^55^. For data preprocessing, 10X-transformed data were imported only considering genes that are found in at least 3 cells and cells that expressed at least 10 genes. Further filtering of cells was performed for each data set by excluding cells that had less than 500 unique feature counts and otherwise were within the 97.5%-percentiles with regard to the unique feature counts, the total number of molecules detected within each cell and the percentage of mitochondrial gene expression to exclude low quality cells and possible multiplets. After filtering, we obtained a total set of 9647 cells with 21341 features for TAT-CRE, and 8108 cells with 19607 features for AD-CRE, indicating considerable comparability of the two samples.

After quality control, data were normalized, scaled and analyzed for variable features using the function SCTtransform within Seurat with both data sets integrated based on the canonical correlation analysis (CAA) anchor-based integration method to account for possible batch effects. Afterwards a principal component analysis (PCA) was performed on the integrated data set with the number of considered PCA determined by the Elbow-method, leaving 21 PCA-components for subsequent analysis. Individual cell clustering was obtained using the Leiden-clustering algorithm^56^ with subsequent application of Uniform manifold approximation and projection (UMAP) for data visualization. To determine the major cell types of the identified clusters, we performed automatic cell type detection by SCtype^57^ using the cell type atlases “Lung” and “Immune system” with subsequent comparison. To identify the status of malignancy indicated as cancerous and non-malignant, the epithelial annotated cells are sub-classified on two levels using the SCtype tool^57^. In detail, SCtype determined the cancerous cells in a certain cell type above the median single-nucleotide variation (SNV) in the cancer consensus genes across all cells within the sample, and incorporated aneuploidy using a Bayesian segmentation approach^57,58^. Differential genes expression analysis between the different conditions for each of the identified cell clusters was performed in Seurat focusing on the top 20 differentially expressed genes. The researcher performing the scRNA Seq analysis was not-blinded.

### Genotyping PCR

Eartag material was lysed using 100 µL lysis buffer (5 ∙ 10^−4^M EDTA, 0.025 M NaOH) and incubating for 60 min 800 rpm 100 °C and neutralized by adding 100 µL neutralization buffer (40 mmol TRIS, pH 5). 2 µL of extracts will be used in the PCR protocol using the GoTaq G2 Green system (M7822) according to manufacturer’s advice. Genotyping was performed in a non-blinded manner. Trp53 genotyping PCR runs for 95 °C 3 min, and 38 cycles of 95 °C for 30 sec, 54 °C for 30 sec, 72 °C for 1 min., and finalize with 10 min at 72 °C. Primer sequences for the Trp53 genotyping PCR are noted 5′-3′: Trp53-fwd AAGGGGTATGAGGGACAAGG; Trp53-rev GAAGACAGAAAAGGGGAGGG (product: 584 bp). Kras^G12D^ genotyping PCR runs for 95 °C 3 min, and 38 cycles of 95 °C for 30 sec, 58 °C for 30 sec, 72 °C for 1 min., and finalize with 10 min at 72 °C. Primer sequences for the Kras^G12D^ genotyping PCR are noted 5′-3′: Kras-fwd CCATGGCTTGAGTAAGTCTGCA; Kras-rev CGCAGACTGTAGAGCAGCG (product: 550 bp). Genotyping results are used to identify which mice were carrying each of the floxed alleles (Supplementary Fig. 4).

### Nested PCR

We investigated the duration of AD-CRE persisting in the body of inhaled mice in order to potentially re-classify them as GE-biosafety level S1 after AD-CRE application. We performed a nested PCR protocol with a sensitivity of 50 PFU to detect AD-CRE in stool samples (Supplementary Fig. 5a). As controls, we also took stool samples prior to inhalation and stool samples of TAT-CRE inhaled mice, as well. AD-CRE DNA is detectable in four out of five stool samples 24 h after AD-CRE inhalation but not after 48 h (Supplementary Fig. 5b, c).

The nested PCR was performed with initial denaturation at 94 °C for 2 min, and 30 cycles of 94 °C for 20 sec, 72 °C for 60 sec, and finalize with 10 min at 72 °C. 1 µL of sample extract was be used in a 20 µL PCR reaction. Primer sequences for the first PCR are noted 5′-3′: Ad5-I-fwd CAGCACGGGTAATATGGGTGTTCTGGCG; Ad5-I-rev TGCGGTGGTGGTTAAATGGGTTGACGTTG. The product of the first PCR was diluted 1:10. A volume 0.5 µL is used for second PCR resulting in a final dilution of 1:400 in a 20 µL PCR reaction. The second PCR ran at 94 °C for 2 min, and 30 cycles at 94 °C for 20 sec, 58 °C for 30 sec, 72 °C for 30 sec, and finalized with 10 min at 72 °C. Primer sequences for the second PCR are noted 5′-3′: Ad5-II-fwd GAAACACAGAGCTTTCATACCA; Ad5-II-rev TGTAGTCGTAGGTGTTTGGG. The PCR protocol was performed with the GoTaq® reaction system (Promega) according to manufacturer’s advice in a non-blinded manner.

### Ethics

We have complied with all relevant ethical regulations for animal use. Animal studies were carried out in accordance to the recommendations of the Federation of European Laboratory Animal Science Association (FELASA) and the Society of Laboratory Animal Science (GV-SOLAS). The study protocol was approved by the local Ethics Committee of Animal experiments and the Landesamt für Natur, Umwelt und Verbraucherschutz of North Rhine-Westphalia in Germany (LANUV; 81-02.04.2020.A026). Murine cell isolations were approved by the local Ethics Committee of Animal experiments of the Friedrich-Alexander University Erlangen-Nuremberg (Germany) under the approval number TS-8/2023 Exp Med I.

### Statistics and reproducibility

Statistical analyses and visualization of the data were performed in R and in GraphPad Prism (Graphpad Software, Inc.). Sample sizes were chosen based on included and approved animal numbers. Cells in scRNA Seq exhibiting high mitochondrial and ribosomal gene content were excluded. Number of biological replicates was defined in the figure legend and based on the included and approved animal numbers. Male and female mice have been randomized into the groups before tumor induction. Analysis of parameters in the investigated groups has been performed blinded if possible. Whether analysis was performed in a blinded or not blinded manner is indicated in the text. The statistical methods used for each analysis are described in figure legends and in the text. Statistical significance is indicated as follows: *p*value  <  0.1 is not significant (ns), *pvalue  ≤  0.05, **pvalue  ≤  0.01, ***pvalue  ≤  0.001 and ****pvalue ≤ 0.0001.

### Reporting summary

Further information on research design is available in the Nature Portfolio Reporting Summary linked to this article.

## Supplementary information

Supplementary Information

Description of Additional Supplementary Materials

Supplementary Data

Reporting Summary

## Data Availability

Single-cell RNA-seq data are deposited in the Gene Expression Omnibus (GSE294262). All other data are available from the corresponding author on reasonable request. The source data are available in Supplementary Data 1 and annotated with sex: male (blue), female (violet).
