# Supplementary material for: TAT-CRE inhalation enables tumor induction corresponding to adenoviral Cre-recombinase in a lung cancer mouse model
Source: Commun Biol. 2025 May 13;8:741. doi: 10.1038/s42003-025-08146-0 (PMC12075843; doi:10.1038/s42003-025-08146-0)
Supplement: Supplementary file 1 — Supplementary Information [file 42003_2025_8146_MOESM1_ESM.pdf]

Supplementary Figures

TAT-CRE inhalation enables tumor induction corresponding to adenoviral Cre-recombinase in a lung cancer mouse model

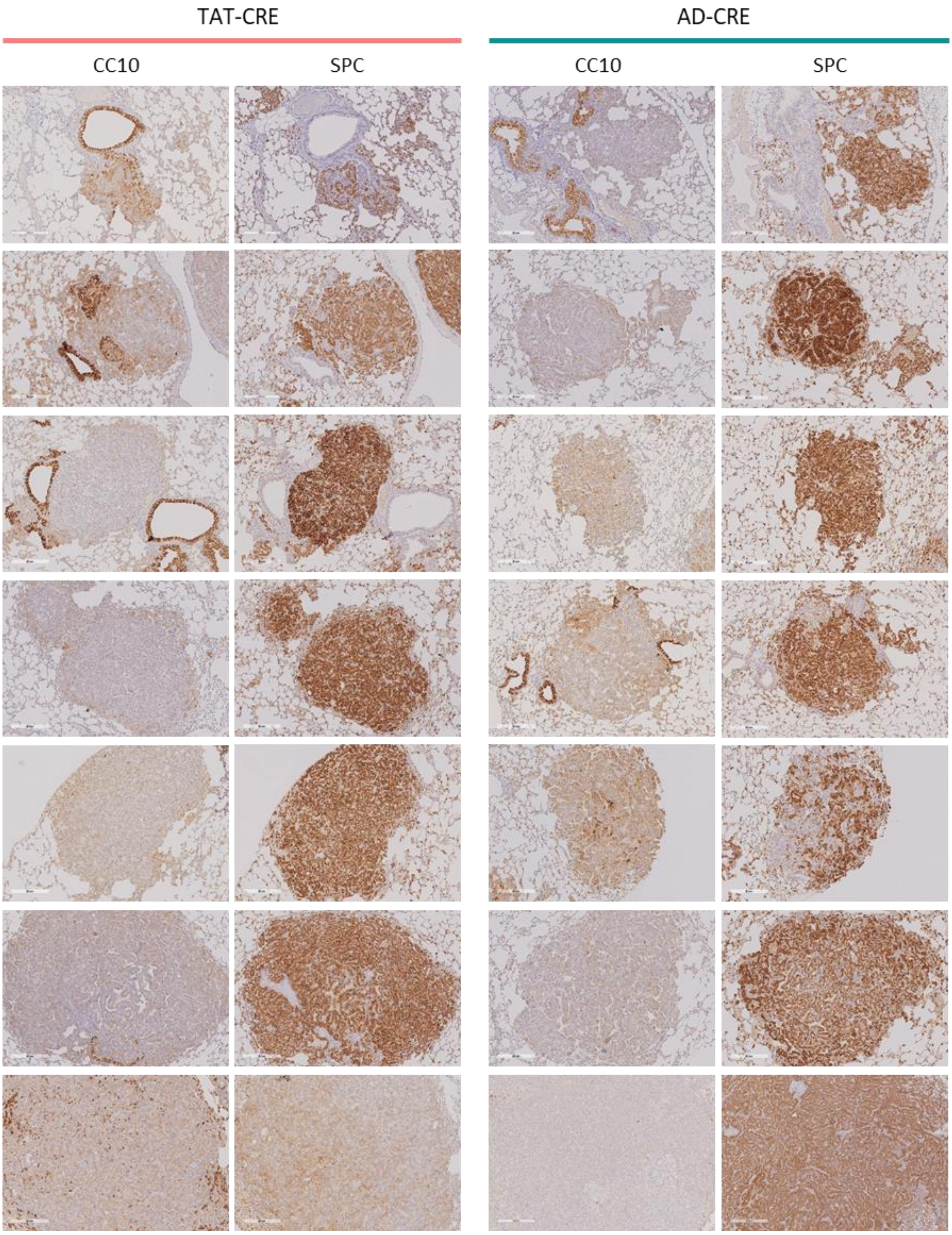

Supplementary Figure 1. TAT-CRE and AD-CRE induced lung tumors expressed CC10 and SPC.
Lung adenocarcinomas induced by TAT-CRE and AD-CRE inhalation were analyzed for pulmonary markers CC10 and SPC. A tumor lesion was defined by a minimal diameter of 100 µm. Representative IHC stain of CC10 and SPC, magnification 10x. Bars indicate 200 µm.

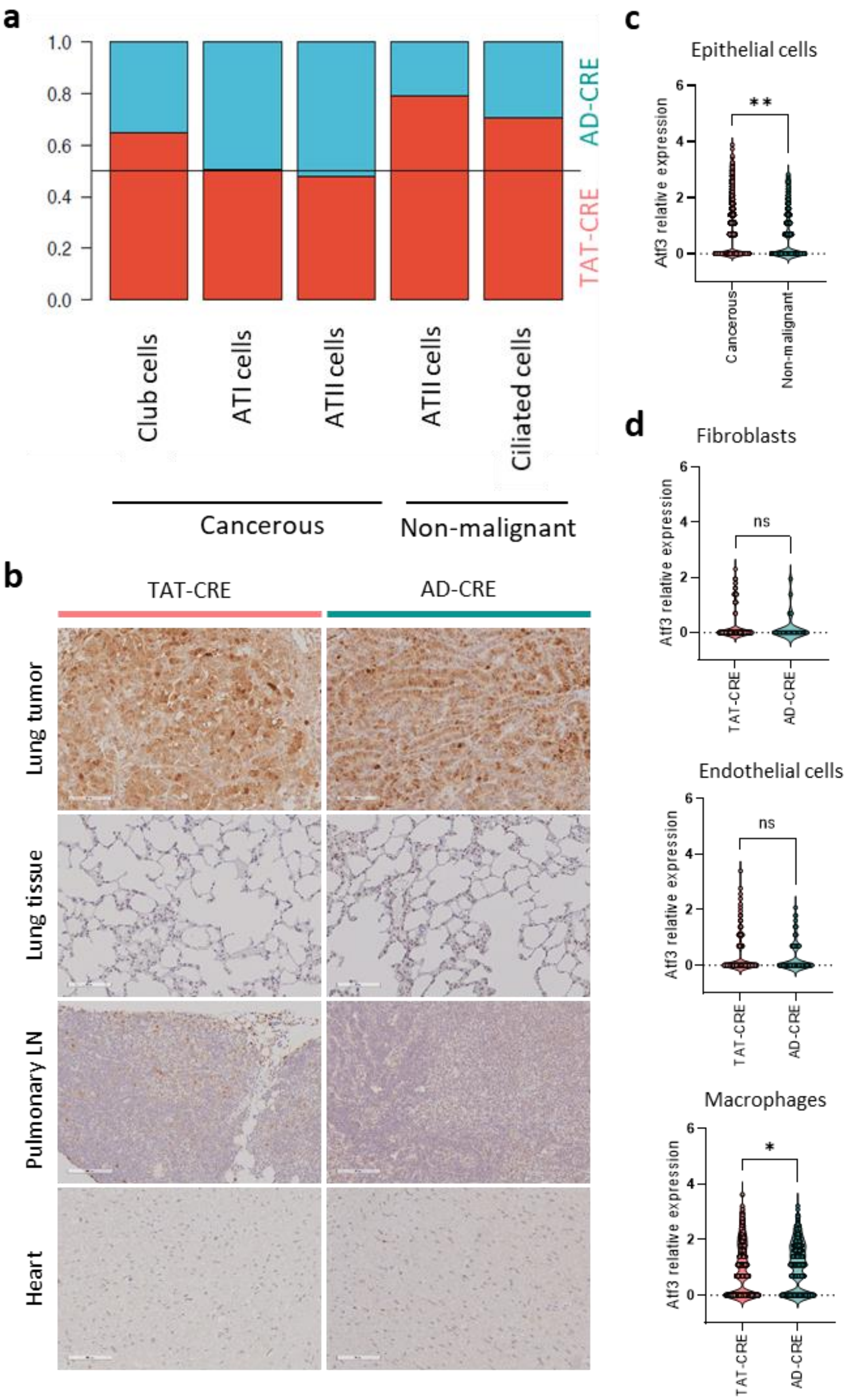

Supplementary Figure 2. TAT-CRE and AD-CRE induced recombination *in vivo*.

Lung adenocarcinomas induced by TAT-CRE and AD-CRE inhalation were analyzed for parameters of recombination. **a** Stacked bar plot showing the scRNA Seq based fraction per condition (TAT-CRE vs AD-CRE) among pulmonary cancerous and non-malignant cell types: AT1 (alveolar type 1) cells, AT2 (alveolar type 2) cells, Club cells and ciliated cells. **b** pERK1/2 was analyzed by IHC as surrogate for

mutated *Kras* activated signaling. Reference tissues located in the proximity of lung tumors were also analyzed comprising normal lung tissue, pulmonary lymph nodes (LN) and the heart. Representative IHC stain of pERK1/2, magnification 20x. Bars indicate 100 µm. **c** Differentially expressed *Arf3* transcripts in cancerous and non-malignant pulmonary cell types of cumulative CRE induced lung adenocarcinoma samples analysed by scRNA Seq. **d** Differentially expressed *Arf3* transcripts in fibroblasts, endothelial cells and macrophages of TAT-CRE and AD-CRE induced lung adenocarcinoma samples analysed by scRNA Seq. p-values were calculated using an unpaired two-sided t-test. Data is shown as mean ± SD. P-values ≤ 0.05 are considered as significant.

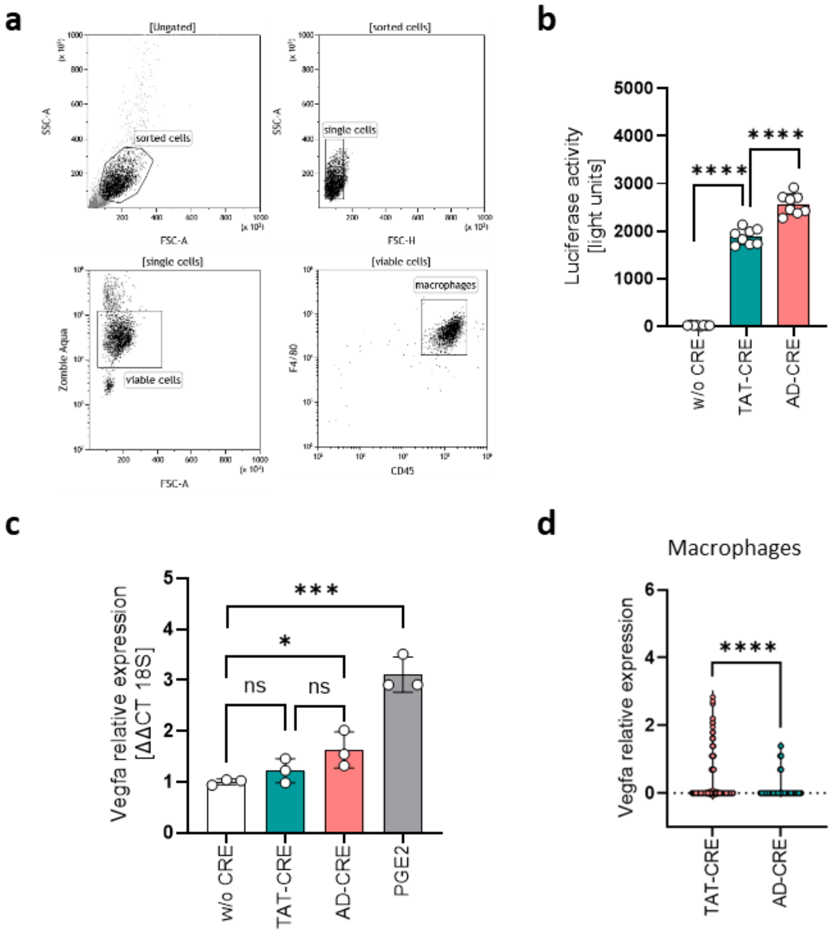

Supplementary Figure 3. Cre-recombination in macrophages enabled Vegfa expression.

a Macrophages were isolated from a Cre-reporter mouse with a luciferase gene by magnetic activated cell sorting. **b** Luciferase activity was determined upon Cre-recombination (w/o, without). **c** Real-time PCR was used to determine the relative expression level of Vegfa in *B6.129-Kras*^{tm4Tyj} *Trp53*$^{tm1Brn/J}$ macrophages upon indicated conditions. Prostaglandin E2 (PGE2) stimulation serves as a positive control. Gapdh was used for normalization followed by the ΔΔCT-method. **d** *Vegfa* transcripts in macrophages of TAT-CRE and AD-CRE induced lung adenocarcinoma samples analysed by scRNA Seq. p-values were calculated using an unpaired two-sided t-test. Data is shown as mean ± SD. P-values ≤ 0.05 are considered as significant.

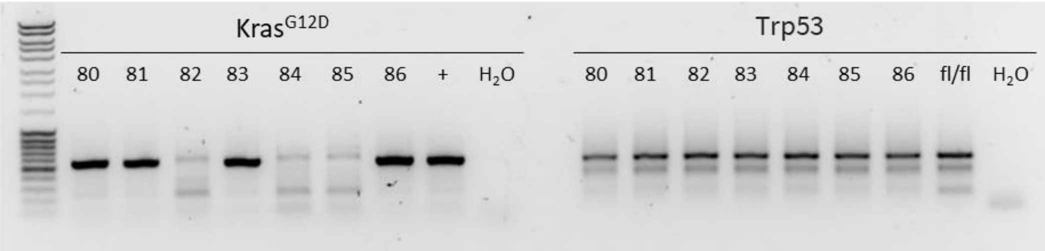

Supplementary Figure 4. Genotyping of B6.129-Kras^{tm4Tyj} Trp53$^{tm1Brn/J}$.
Genotyping PCR was performed based on DNA isolated from eartag material, indicating the floxed transgenic DNA regions leading to the expression of Kras^{G12D} and functional depletion of p53 after Cre-recombinase treatment.

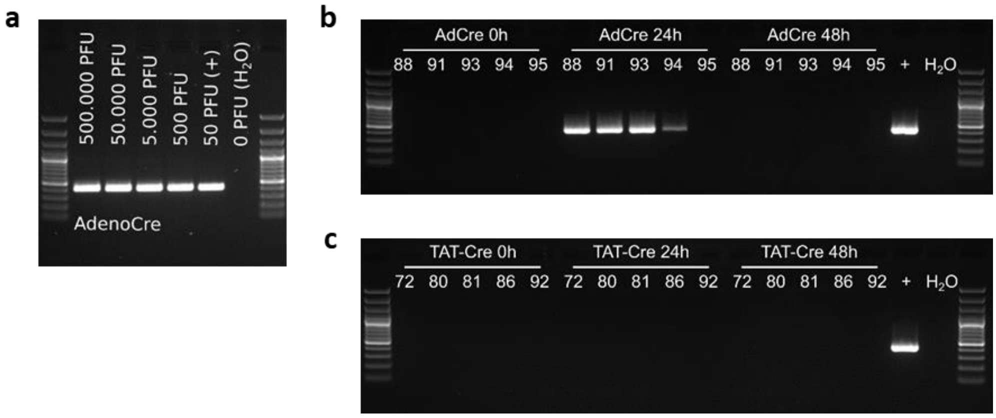

Supplementary Figure 5. Adenovirus detection in feces of Cre-recombinase inhaled mice.
A nested PCR was performed based on DNA isolated from stool samples of inhaled mice to detect the presence of the adenoviral construct. **a** Sensitivity of the nested PCR has been determined based on diluted AD-CRE added to stool samples of control mice. **b** DNA isolated from stool samples of AD-CRE inhaled mice was analyzed using a nested PCR. **c** DNA isolated from stool samples of TAT-CRE inhaled mice was analyzed using a nested PCR.
