# Supplementary material for: TAT-CRE inhalation enables tumor induction corresponding to adenoviral Cre-recombinase in a lung cancer mouse model
Source: Commun Biol. 2025 May 13;8:741. doi: 10.1038/s42003-025-08146-0 (PMC12075843; doi:10.1038/s42003-025-08146-0)
Supplement: Supplementary file 2 — Description of Additional Supplementary Materials [file 42003_2025_8146_MOESM2_ESM.pdf]

Description of Additional Supplementary Files

File name: Supplementary Data

Description: The source data are available in Supplementary Data 1 and annotated with sex: male (blue), female (violet).
