# Supplementary material for: TAT-CRE inhalation enables tumor induction corresponding to adenoviral Cre-recombinase in a lung cancer mouse model
Source: Commun Biol. 2025 May 13;8:741. doi: 10.1038/s42003-025-08146-0 (PMC12075843; doi:10.1038/s42003-025-08146-0)
Supplement: Supplementary file 4 — Reporting Summary [file 42003_2025_8146_MOESM4_ESM.pdf]

nature portfolio

Corresponding author(s): Lydia Meder

Last updated by author(s): Apr 14, 2025

Reporting Summary

Nature Portfolio wishes to improve the reproducibility of the work that we publish. This form provides structure for consistency and transparency in reporting. For further information on Nature Portfolio policies, see our Editorial Policies and the Editorial Policy Checklist.

Statistics

For all statistical analyses, confirm that the following items are present in the figure legend, table legend, main text, or Methods section.

n/a	Confirmed	
☐	☒	The exact sample size (*n*) for each experimental group/condition, given as a discrete number and unit of measurement
☐	☒	A statement on whether measurements were taken from distinct samples or whether the same sample was measured repeatedly
☐	☒	The statistical test(s) used AND whether they are one- or two-sided<br>*Only common tests should be described solely by name; describe more complex techniques in the Methods section.*
☐	☒	A description of all covariates tested
☐	☒	A description of any assumptions or corrections, such as tests of normality and adjustment for multiple comparisons
☐	☒	A full description of the statistical parameters including central tendency (e.g. means) or other basic estimates (e.g. regression coefficient) AND variation (e.g. standard deviation) or associated estimates of uncertainty (e.g. confidence intervals)
☐	☒	For null hypothesis testing, the test statistic (e.g. *F*, *t*, *r*) with confidence intervals, effect sizes, degrees of freedom and *P* value noted<br>*Give P values as exact values whenever suitable.*
☒	☐	For Bayesian analysis, information on the choice of priors and Markov chain Monte Carlo settings
☒	☐	For hierarchical and complex designs, identification of the appropriate level for tests and full reporting of outcomes
☒	☐	Estimates of effect sizes (e.g. Cohen's *d*, Pearson's *r*), indicating how they were calculated

Our web collection on statistics for biologists contains articles on many of the points above.

Software and code

Policy information about availability of computer code

Data collection

Single-cell RNA sequencing of lung tumors was performed by the Singleron Biotechnologies GmbH in Cologne, Germany. A piece of the tumor tissue with a diameter of 3-5 mm was embedded in Sample Preparation Buffer (Singleron Biotechnologies) stable for up to 72 hours prior processing. Single cell suspension from tumor tissue were processed in sCelLiVETM Tissue Dissociation Buffer. Then, single cells were loaded into the microfluidic SCOPE-chipTM and cell identifying tags were flowed in and allowed to settle in the wells on top of the cells. mRNA from each lysed cell was hybridized to the barcode sequences on the bead in the same well. Then, reverse transcription of primed RNA and cDNA amplification take place. The amplified cDNA was fragmented, adapter ligated and amplified to construct a sequencing library suitable for the Illumina sequencing platform.

Data analysis

The analysis of the scRNA-seq data was performed within R version 4.3.2 using Seurat-package 5.0.153. For data preprocessing, 10X-transformed data were imported only considering genes that are found in at least 3 cells and cells that expressed at least 10 genes. Further filtering of cells was performed for each data set by excluding cells that had less than 500 unique feature counts and otherwise were within the 97.5%-percentiles with regard to the unique feature counts, the total number of molecules detected within each cell and the percentage of mitochondrial gene expression to exclude low quality cells and possible multiplets. After filtering, we obtained a total set of 9647 cells with 21341 features for TAT-CRE, and 8108 cells with 19607 features for AD-CRE, indicating considerable comparability of the two samples.
After quality control, data were normalized, scaled and analyzed for variable features using the function SCTtransform within Seurat with both data sets integrated based on the canonical correlation analysis (CAA) anchor-based integration method to account for possible batch effects. Afterwards a principal component analysis (PCA) was performed on the integrated data set with the number of considered PCA determined by the Elbow-method, leaving 21 PCA-components for subsequent analysis. Individual cell clustering was obtained using the Leiden-clustering algorithm54 with subsequent application of Uniform manifold approximation and projection (UMAP) for data visualization. To determine the major cell types of the identified clusters, we performed automatic cell type detection by SCtype55 using the cell type atlases “Lung” and “Immune system” with subsequent comparison. To identify the status of malignancy indicated as cancerous and non-malignant, the epithelial

annotated cells are sub-classified on two levels using the SCtype tool55. In detail, SCtype determined the cancerous cells in a certain cell type above the median single-nucleotide variation (SNV) in the cancer consensus genes across all cells within the sample, and incorporated aneuploidy using a Bayesian segmentation approach55,56. Differential genes expression analysis between the different conditions for each of the identified cell clusters was performed in Seurat focusing on the top 20 differentially expressed genes. The researcher performing the scRNA Seq analysis was not-blinded.

For manuscripts utilizing custom algorithms or software that are central to the research but not yet described in published literature, software must be made available to editors and reviewers. We strongly encourage code deposition in a community repository (e.g. GitHub). See the Nature Portfolio guidelines for submitting code & software for further information.

Data

Policy information about availability of data

All manuscripts must include a data availability statement. This statement should provide the following information, where applicable:

- Accession codes, unique identifiers, or web links for publicly available datasets
- A description of any restrictions on data availability
- For clinical datasets or third party data, please ensure that the statement adheres to our policy

Single-cell RNA-seq data are deposited in the Gene Expression Omnibus (GSE294262). The other data are available within Supplementary files or available from the authors upon request. The source data are available in Supplementary Data 1.

Research involving human participants, their data, or biological material

Policy information about studies with human participants or human data. See also policy information about sex, gender (identity/presentation), and sexual orientation and race, ethnicity and racism.

Reporting on sex and gender	NA
Reporting on race, ethnicity, or other socially relevant groupings	NA
Population characteristics	NA
Recruitment	NA
Ethics oversight	NA

Note that full information on the approval of the study protocol must also be provided in the manuscript.

Field-specific reporting

Please select the one below that is the best fit for your research. If you are not sure, read the appropriate sections before making your selection.

☒ Life sciences ☐ Behavioural & social sciences ☐ Ecological, evolutionary & environmental sciences

For a reference copy of the document with all sections, see nature.com/documents/nr-reporting-summary-flat.pdf

Life sciences study design

All studies must disclose on these points even when the disclosure is negative.

Sample size	Sample sizes are chosen based on included and approved animal numbers.
Data exclusions	Cells in scRNA Seq exhibiting high mitochondrial and ribosomal gene content were excluded.
Replication	Replicates have been performed based on the included and approved animal numbers.
Randomization	Male and female mice have been randomized into the groups before tumor induction.
Blinding	Analysis of parameters in the investigated groups has been performed blinded if possible. Whether analysis was performed in a blinded or not blinded manner is indicated in the text.

Reporting for specific materials, systems and methods

We require information from authors about some types of materials, experimental systems and methods used in many studies. Here, indicate whether each material, system or method listed is relevant to your study. If you are not sure if a list item applies to your research, read the appropriate section before selecting a response.

Materials & experimental systems

n/a	Involved in the study
☐	☒ Antibodies
☒	☐ Eukaryotic cell lines
☒	☐ Palaeontology and archaeology
☐	☒ Animals and other organisms
☒	☐ Clinical data
☒	☐ Dual use research of concern
☒	☐ Plants

Methods

n/a	Involved in the study
☒	☐ ChIP-seq
☐	☒ Flow cytometry
☒	☐ MRI-based neuroimaging

Antibodies

Antibodies used

IHC: Primary Antibodies used: KI-67 (D3B5, Cell Signaling, #12202, 1:2000, TRS6 1 minute), cleaved Caspase 3 (polyclonal, Cell Signaling, #9661, 1:400, TRS6 1 minute), CD31 (7O7K3, Thermo Fischer Scientific, MA5-37858, 1:400, TRS6 1 minute, AB-Block), uteroglobin (also known as CC10, polyclonal, Thermo Fischer Scientific, PA5-102469, 1:20000, TRS6 10 minutes, AB-Block), surfactant protein C (SPC, polyclonal, Thermo Fischer Scientific, PA5-102493, 1:1000, Citrate 1 minute), claudin-3 (polyclonal, Thermo Fischer Scientific, # 34-1700, 1:500, Citrate 1 minute), phospho-p44/42 MAPK (Erk1/2) (Thr202/Tyr204) (20G11, Cell Signaling, #4376, 1:1000, TRS6 1 minute).
FACS: CD45 (FITC, 30-F11), CD11c (PE-Dazzle594, N418), CD11b (PE-Cy7, M1/70), F4/80 (Alexa Fluor 700, BM8), VEGFR2 (PerCP-Cy5.5, 89B3A5), Rat IgG2aK (PerCP-Cy5.5, Alexa Fluor 700), PE-Dazzle594 Armenian Hamster IgG (PE-Dazzle594), Rat IgG2bK (FITC, PE-Cy7).

Validation

The antibody validation occurred as follows. CD31 and claudin 3 antibodies have been validated by the distributor using genomic knock-out. CC10, SPC, and KI-67 have been validated by the distributor on control tissue, testis, lungs and colon, respectively. Cleaved caspase-3 was validated by the distributor using Cytochrome C stimulation of cancer cells. We have validated all antibodies for specific binding pattern and optimal concentration on murine lung tissue and if applicable, also on human lung tissue and in representative cells. Different antibody concentrations have been tested for each antibody and the most descriptive one was chosen.

Animals and other research organisms

Policy information about studies involving animals; ARRIVE guidelines recommended for reporting animal research, and Sex and Gender in Research

Laboratory animals

genetically engineered mouse model B6.129-Krastm4Tyj Trp53tm1Brn/J was used to induce lung adenocarcinoma

Wild animals

NA

Reporting on sex

Male and female mice with at least 20 g body weight have been included and randomized into the groups.

Field-collected samples

NA

Ethics oversight

Animal studies were carried out in accordance to the recommendations of the Federation of European Laboratory Animal Science Association (FELASA) and the Society of Laboratory Animal Science (GV-SOLAS). The study protocol was approved by the local Ethics Committee of Animal experiments and the Landesamt für Natur, Umwelt und Verbraucherschutz of North Rhine-Westphalia in Germany (LANUV; 81-02.04.2020.A026). Murine cell isolations were approved by the local Ethics Committee of Animal experiments of the Friedrich-Alexander University Erlangen-Nuremberg (Germany) under the approval number TS-8/2023 Exp Med I.

Note that full information on the approval of the study protocol must also be provided in the manuscript.

Plants

Seed stocks

NA

Novel plant genotypes

NA

Authentication

NA

Flow Cytometry

Plots

Confirm that:

☒ The axis labels state the marker and fluorochrome used (e.g. CD4-FITC).

☒ The axis scales are clearly visible. Include numbers along axes only for bottom left plot of group (a 'group' is an analysis of identical markers).

☒ All plots are contour plots with outliers or pseudocolor plots.

☒ A numerical value for number of cells or percentage (with statistics) is provided.

Methodology

Sample preparation	Lungs of mice were harvested at a progressed tumor stage at maximum of 14 weeks after inhalation. Cells were isolated using 40 µm cell strainers (BD Falcon) after mechanical dissociation. ACK lysis buffer (Life Technologies) was applied 10 min at room temperature to lyse red blood cells. Cells were washed with PBS prior to staining. Cell suspensions are stained for 30 min at 4 °C using primary antibodies and isotype controls
Instrument	Flow cytometry measurements were performed on a Gallios 10/3 (Beckman Coulter).
Software	Data was analyzed using Kaluza (Beckman Coulter).
Cell population abundance	The analyzed macrophage population has a purity of more than 90 % in the parent population.
Gating strategy	Cell viability was assessed by staining with Zombie Aqua. Dead cells positive for Zombie Aqua are excluded. CD45 was used as a marker for immune cells and CD11b was used to identify immune cells of the myeloid lineage. CD11c was used as a marker for dendritic cells and alveolar macrophages, and in a next step F4/80 positivity was needed to discriminate macrophages from other myeloid cells such as eosinophils.

☒ Tick this box to confirm that a figure exemplifying the gating strategy is provided in the Supplementary Information.
